# PPR14 Interacts With PPR-SMR1 and CRM Protein Zm-mCSF1 to Facilitate Mitochondrial Intron Splicing in Maize

**DOI:** 10.3389/fpls.2020.00814

**Published:** 2020-06-12

**Authors:** Hong-Chun Wang, Zongliang Chen, Yan-Zhuo Yang, Feng Sun, Shuo Ding, Xiu-Lan Li, Chunhui Xu, Bao-Cai Tan

**Affiliations:** Key Laboratory of Plant Development and Environment Adaptation Biology, Ministry of Education, School of Life Sciences, Shandong University, Qingdao, China

**Keywords:** interaction, intron splicing, maize, mitochondria, pentatricopeptide repeat (PPR) protein, seed development

## Abstract

In plants, splicing of organellar group II introns involves numerous nucleus-encoded *trans-*factors. But, how these *trans-*factors function and interact is not well understood. Here we report the function of a pentatricopeptide repeat (PPR) protein PPR14 and its physical relationship with other splicing factors in mitochondria. Null mutations of *PPR14* severely arrest the embryo and endosperm development, causing an empty pericarp phenotype. PPR14 is required for the splicing of *NADH dehydrogenase 2* (*nad2*) intron 3 and *nad7* introns 1 and 2 in mitochondria. The absence of *nad2* and *nad7* transcripts leads to disruption of the mitochondrial complex I assembly and abolishes its NADH dehydrogenase activity. This is accompanied with increased levels of other mitochondrial complexes and elevated expression of the alternative oxidase proteins. As the function of PPR14 overlaps with PPR-SMR1 and the CRM-domain containing protein Zm-mCSF1, we tested their interactions. Protein-protein interaction analysis indicated that PPR14 interacts with PPR-SMR1 and Zm-mCSF1, suggesting that these three proteins may form a complex. As PPR proteins and CRM-domain containing proteins have many members in mitochondria and chloroplasts, we propose that organellar group II intron splicing is probably mediated by a dynamic complex that includes different PPR and CRM proteins in plants.

## Introduction

In higher plants, most genes of the endosymbiont are either lost or transferred to nucleus during evolution (Martin, 2003; Huang et al., 2005). Consequently, the mitochondrial genome only retains about 60 identified genes. The maize mitochondrial genome was reported to include 58 identified genes encoding 3 ribosomal RNAs (rRNAs), 22 tRNAs and 33 known proteins involved in respiratory chain directly or indirectly (Clifton et al., 2004). The expression of mitochondrial genes is regulated at transcriptional, posttranscriptional, translational, and posttranslational levels. Among them, post-transcriptional processes, which have been reported to play predominant roles, include RNA cleavage, RNA stabilization, RNA editing, RNA splicing of group I and group II introns, and RNA maturation (Hammani and Giegé, 2014). In maize, the mitochondrial genome contains 22 group II introns. The majority are *cis*-splicing introns, which are excised from single precursor RNAs. Six of them are *trans-*splicing introns and widely scattered in the genome along with their up- or down-stream exons due to mitochondrial DNA rearrangements. These exon-intron segments are transcribed independently and exons are ligated together through *trans*-splicing.

Group I and group II introns are commonly found in organelle genomes of plants, fungi, and protists, and also in prokaryotes. Furthermore, group I introns are common in nuclear rRNA genes of unicellular eukaryotes (Nielsen and Johansen, 2009; Nyati et al., 2013), whereas group II introns have not been identified in nuclear genes of any eukaryotes (Bonen, 2011). In higher plants, most introns of organelle genomes are group II introns. Group I and group II introns have distinctive secondary structures, which determine the classification of introns. Group I introns are composed of ten helices (P1-P10), which form three helical stacks (Nielsen and Johansen, 2009). Unlike group I introns, group II introns fold into six structural domains (DI-DVI) and highly conserved domain V forms the catalytic core of intron splicing. Such intron structure model has been supported by crystal structures of two group II introns: a mitochondrial intron *P.li.LSUI2* from the brown algae *Pylaiella littoralis* (Robart et al., 2014) and a bacterium intron from *Oceanobacillus iheyensis* (Toor et al., 2008). Some group I and group II introns are ribozymes *in vitro*, although none in higher plant mitochondria or plastids have been demonstrated to have autocatalytic activity. These two classes of introns are spliced via two transesterification reactions, but the splicing mechanisms are different. In the first step of classical group II intron splicing, the 2′ OH of a bulged adenosine in the domain VI (DVI) near 3′ end of the intron acts as nucleophile to attack 5′ splice site. After the first step, a 5′ dissociative exon and a lariat RNA containing the 3′ exon are produced. In the second transesterification step, the 3′ OH of the upstream exon acts as the nucleophile to attack 3′ splice site and generate ligated exons and intron lariat (Brown et al., 2014).

According to previous studies, splicing of plant organellar group II introns requires various protein cofactors *in vivo*. In higher plants, the mitochondrial group II intron splicing factors identified so far mainly include several families: maturase, PORR (Plant Organellar RNA Recognition) domain family, RCC (Regulator of Chromosome Condensation) protein, mTERF (mitochondrial transcription termination factor), DEAD-box RNA helicase, CRM (chloroplast RNA splicing and ribosome maturation) proteins, and PPR (pentatricopeptide repeat) proteins. In bacteria, maturases encoded within group II introns facilitate their own intron splicing. While most organellar introns in plants have lost their specific maturases (Schmitz-Linneweber et al., 2015) and only a single maturase-related (MatR) protein encoded within the fourth intron of NADH dehydrogenase 1 (*nad1*) has been retained in angiosperm mitochondria (Wahleithner et al., 1990). Recent studies have shown that MatR is required for the splicing of *nad1* intron 4 and several other group II introns *in vivo* (Sultan et al., 2016). In addition to MatR, mitochondria also contain four nucleus-encoded maturases (nMAT 1–4) in Arabidopsis. Among them, nMAT1 and nMAT2 are required for splicing of three distinct mitochondrial introns, respectively, while nMAT4 is implicated in the splicing of 8 mitochondrial introns (Keren et al., 2009, 2012; Cohen et al., 2014). Genetic studies indicate that a PORR protein WTF9 functions in the group II intron splicing of *rpl2* (encoding an essential ribosomal protein) and *ccmFc* (encoding a component of the cytochrome *c* maturation system) in Arabidopsis mitochondria (des Francs-Small et al., 2012). The RCC proteins consist of seven tandem repeats of a conserved 50-amino-acid domain. A RCC1 family protein RUG3 was shown to facilitate the splicing of *nad2* in mitochondria of Arabidopsis (Kühn et al., 2011). Recent studies suggest that mTERFs, a nucleus-encoded DNA/RNA-binding protein family, is required for the regulating of organellar transcription. A mitochondria-localized mTERF15 is involved in *nad2* intron 3 splicing in Arabidopsis (Hsu et al., 2014). One member of the DEAD-box RNA helicases, PMH2 (Putative Mitochondrial Helicase 2), was reported to play important roles in efficient splicing of 15 mitochondrial introns in Arabidopsis (Köhler et al., 2010).

Similarly, a CRM-domain containing protein (mCSF1, mitochondrial CAF-like splicing factor 1) is also required for splicing of numerous group II introns in Arabidopsis mitochondria (Zmudjak et al., 2013). CRM-domain proteins are characterized by an RNA-binding domain, which is similar to a conserved bacterial YhbY domain (Barkan et al., 2007). PPR proteins are defined as 2–26 tandem repeats of a loosely conserved 35-amino-acid motif (Small and Peeters, 2000). They are prevalent in land plants and classified into two classes (P and PLS) based on motif composition (Lurin et al., 2004; Schmitz-Linneweber and Small, 2008). Recent studies have shown that most PPR proteins are required for the post-transcriptional processing events in plastids and mitochondria (Barkan and Small, 2014). PPR proteins involved in the splicing of specific mitochondrial transcripts include OTP43 (de Longevialle et al., 2007), ABO5 (Liu et al., 2010), BIR6 (Koprivova et al., 2010), SLO3 (Hsieh et al., 2015), MTL1 (Haïli et al., 2016), TANG2, and OTP439 (des Francs-Small et al., 2014) in Arabidopsis and EMP16 (Xiu et al., 2016), Dek35 (Chen et al., 2017), Dek2 (Qi et al., 2017), and PPR-SMR1 (Chen et al., 2019) in maize. All these splicing cofactors belong to the P-type PPR proteins. Loss-of-function mutations in these PPR splicing factors cause retarded growth or defective seed development, indicating that PPR proteins are required for plant growth and embryogenesis. However, little is known about the molecular mechanisms of PPR proteins involved in intron splicing.

To elucidate the mechanism by which group II introns are spliced in mitochondria, we determined the molecular functions of a mitochondrion-targeted P-type PPR protein containing 14 PPR motifs (GRMZM2G106384), thus named PPR14, in maize. Loss-of-function mutation in *PPR14* causes the arrest of embryo and endosperm development, leading to an empty pericarp (emp) phenotype. Functional analysis indicates that PPR14 is required for the splicing of *nad2* intron 3 and *nad7* introns 1 and 2 in mitochondria. Lack of these splicing events results in the absence of functional Nad2 and Nad7 proteins that are essential for complex I assembly. Consequently, it disrupts the mitochondrial respiratory chain and causes defective seed development. In addition, we find that PPR14 can interact with a PPR-SMR protein PPR-SMR1 and a CRM-domain protein Zm-mCSF1. These findings provide insights to the intron splicing mechanism in mitochondria and imply a similar mechanism may exist in chloroplasts as well.

## Materials and Methods

### Plant Materials

Two independent loss-of-function mutant alleles of PPR14 were used in this study, namely *ppr14-1* and *ppr14-2* which were isolated from the UniformMu population by introgressing *Mu*-active lines into the inbred W22 genetic background (McCarty et al., 2005). *Mu* insertion sites of the *ppr14* alleles were confirmed by PCR amplification using *PPR14*-specific primers and *Mu* primers (Tan et al., 2011). Both alleles showed the same *empty pericarp* (*emp*) phenotype and crossed progeny between the alleles produced emp kernels, hence *ppr14-1* was used as a representative allele of PPR14 in most analyses and both alleles were used in functional analyses. The wild-type materials refer to siblings of the *ppr14* mutant. The maize (*Zea mays*) plants were grown in the experimental field under natural conditions.

### DNA Extraction and Linkage Analysis

Maize genomic DNA was isolated by a urea-phenol-chloroform-based extraction method as described previously (Tan et al., 2011). For linkage analysis, the DNA was extracted from individual seedlings and the genotype was determined by PCR analysis using *PPR14*-specific primer 14-F2 and *Mu* primers. The self-crossing ears were checked for the segregation of *emp* mutants. All self-crossing ears of heterozygous *ppr14*/*PPR14* plants segregate *emp* mutants and all self-crossing ears of wild-type do not segregate mutants.

### Light Microscopy of Cytological Sections

Wild-type and mutant kernels were collected from the same ear of a self-pollinated *ppr14-1* heterozygote at 8, 13, and 18 days after pollination (DAP). Sectioning was performed as described previously (Liu et al., 2013). Samples were sectioned at 11-μm thickness under a Microm HM 315. The sections were stained with Johansen’s Safranin O and observed with a Zeiss Scope.A1 microscope.

### Subcellular Localization of PPR14

The first 903 bp of *PPR14* coding region was cloned into Entry vector pENTR/D-TOPO (Invitrogen), then introduced into pGWB5 vector by LR reaction (Invitrogen), generating the PPR14^*N*301^-GFP fusion construct driven by the cauliflower mosaic virus 35S promoter (p*35S*). The construct p*35S*:*PPR14^*N*301^-GFP* was transformed into *Agrobacterium tumefaciens* strain EHA105. Afterward, the Agrobacterium cells containing p*35S*:*PPR14^*N*301^-GFP* construct were infiltrated into epidermal cells of tobacco leaves as described previously (van Herpen et al., 2010). The GFP signals were recorded by a confocal laser microscope (LSM 700, Carl Zeiss) at 23–32 h after infiltration. Mitochondria were labeled by MitoTracker Red (Invitrogen) with the final concentration of 80 nM, and chloroplasts were labeled by chlorophyll autofluorescence. Images were obtained using LSM 700 with GFP (488 nm excitation, 495–555 nm emission), Mito Tracker Red (555 nm excitation, 566–625 nm emission), and chlorophyll autofluorescence (488 nm excitation, 630–730 nm emission).

In addition, the full-length coding region of *PPR14* was cloned into pENTR/D-TOPO (Invitrogen), then introduced into pBI221 vector (A gift from Bai Mingyi, Shandong University) by Gateway site-specific recombination, generating the PPR14-GFP fusion plasmid driven by p*35S*. The construct p*35S*:*PPR14-GFP* and the mitochondrial marker p*35S*:*F1-ATPase-*γ*-RFP* plasmid (Jin et al., 2003) were extracted using a HiSpeed^®^ Plasmid Midi Kit (Qiagen), then introduced into Arabidopsis mesophyll protoplasts at a 1:1 (*PPR14-GFP*:*ATPase-*γ*-RFP*) ratio as described previously (Yoo et al., 2007). After incubation at 22°C in the dark for 25 h, the signals of GFP and RFP fusion proteins were detected by LSM 700 with GFP (488 nm excitation, 495–555 nm emission) and RFP (555 nm excitation, 566–625 nm emission). Free GFP as a negative control was also expressed in protoplasts as described above.

### RNA Extraction, Reverse Transcription PCR, and Quantitative Real-Time PCR

Total RNA was extracted from 100 mg of plant material by using a Qiagen RNeasy^®^ Plant Mini Kit^1^. RNA samples were treated with RNase-free DNase I (NEB^2^) to eliminate DNA contamination. The complete removal of DNA contamination was verified by PCR amplification without reverse transcription. The treated RNA samples were reverse transcribed into cDNAs by using a *TransScript*^®^ One-Step gDNA Removal and cDNA Synthesis SuperMix Kit (TransGen^3^). For analysis of *PPR14* expression in *ppr14* mutant alleles and wild-type seeds, reverse transcription polymerase chain reaction (RT-PCR) was performed with primers 14-F1 and 14-R1 at an annealing temperature of 56°C for 32 cycles. The amplification of maize *Actin* gene (GRMZM2G126010) was used as normalization. The primers are listed in Supplementary Table S1.

For analysis of mitochondrial transcripts in the wild-type and *ppr14-1* mutant, total RNA was isolated and treated as described above. The reverse transcription was also performed as described above. The mitochondrial transcripts abundance was analyzed as described previously (Xiu et al., 2016). The splicing efficiency of mitochondrial group II introns between wild-type and *ppr14* was compared by RT-PCR and quantitative real-time polymerase chain reaction (qRT-PCR). For group II intron splicing analysis, a set of primers were designed to cover 22 mitochondrial introns, respectively. For qRT-PCR analysis, cDNAs were prepared from three independent kernels at 11 DAP. QRT-PCR was performed using the Roche FastStart Essential DNA Green Master on a LightCycler^®^ 96 Instrument (Roche^4^). The amplification of maize elongation factor 1 *alpha* gene (*ZmEF1*α, GRMZM2G153541) was used as normalization (Lin et al., 2014). The primers of mitochondrial transcripts were used as described previously (Liu et al., 2013) and other primers are listed in Supplementary Table S1.

### Blue Native PAGE, in-Gel Complex-I Activity, and Western Blotting Assay

Crude mitochondria were isolated from the embryo and endosperm of maize kernels at 12 DAP as described previously (Meyer et al., 2009). Blue native polyacrylamide gel electrophoresis (BN-PAGE) and complex-I activity assay were carried out as described (Meyer et al., 2009). Western blotting assay was performed according to a previous report (Sun et al., 2015). The primary antibodies against maize Cyt*c*_1_, ATPase α-subunit, and AOX (Xiu et al., 2016), wheat Nad9 (Lamattina et al., 1993), and Arabidopsis Cox2 (Agrisera^5^) were used.

### Yeast Two-Hybrid Assay

Coding sequences of *PPR14*, *PPR-SMR1*, and *Zm-mCSF1* were cloned into the bait (pGBKT7) and prey (pGADT7) vectors (Clontech). The primers used to make these constructs are listed in Supplementary Table S1. The various combinations of GAL4 DNA binding domain (BD) and GAL4 activation domain (AD) constructs were co-transformed into the yeast (*Saccharomyces cerevisiae*) strain Y2HGold (Clontech). Empty vectors were used as negative controls. The other manipulations were carried out according to the user manual (Clontech).

### Bimolecular Fluorescence Complementation Assay

Coding sequence of *PPR14* was cloned into pUC-SPYNE, and *PPR-SMR1* and *Zm-mCSF1* were cloned into pUC-SPYCE, respectively, according to a previous report (Walter et al., 2004). Yellow fluorescent protein (YFP) was split into N-terminus (YFP^*N*^) and C-terminus (YFP^*C*^). The primers used to make these constructs are listed in Supplementary Table S1. All these plasmids were extracted using an EndoFree Maxi Plasmid Kit (TIANGEN). Different combinations of YFP^*N*^ and YFP^*C*^ fusion constructs were co-expressed in Arabidopsis mesophyll protoplasts as described previously (Yoo et al., 2007). The mitochondrial marker F1-ATPase-γ-RFP fusion protein (Jin et al., 2003) was also expressed in these protoplasts. Free YFP^*N*^ and YFP^*C*^ were used as negative controls. The signals of YFP and RFP were recorded by a confocal laser microscope (LSM 880, Carl Zeiss).

### Protein Purification and Pull-Down Analysis

Coding sequences of *PPR14* and *PPR-SMR1* (containing the 6xHis coding sequence at their C terminus) were cloned into the pMAL-c2x vector to generate the fusion constructs with MBP and His tags. The coding sequence of *Zm-mCSF1* containing the 6xHis coding sequence at C terminus was cloned into pGEX4T-1 to generate the fusion construct with GST and His tags. The primers used to make the constructs are listed in Supplementary Table S1. All fusion constructs were then transformed into *Rosetta* strain (*Escherichia coli*). Following protein expression by induction with IPTG, the MBP-PPR14-His, MBP-PPR-SMR1-His, GST-Zm-mCSF1-His, and MBP-His fusion proteins were purified using Ni-NTA agarose (Qiagen) according to the manufacturer’s instructions.

For pull-down assay of three proteins, the GST-Zm-mCSF1-His and GST were bound to glutathione Sepharose 4B (GE Healthcare), respectively. The beads and proteins were washed with TEN100 (20 mM Tris–HCl, pH 7.4, 1 mM EDTA, 100 mM NaCl, and 1 mM PMSF) to remove unbound proteins. Subsequently, MBP-PPR14-His, MBP-PPR-SMR1-His, and MBP-His were added to the two tubes. After incubation, the mixture was washed five times with wash buffer (20 mM Tris–HCl, pH 7.4, 1 mM EDTA, 300 mM NaCl, 0.5% NP40, and 1 mM PMSF). The proteins were released from the beads by boiling in 50 μl 2xSDS-PAGE sample buffer and separated onto a 10% SDS-PAGE gel. The gel was blotted to a NC membrane and analyzed by anti-MBP antibody (NEB, 1:10000 dilution).

### Phylogenetic Analysis and Protein Structure Modeling

The amino-acid sequences of PPR14 and its homologs were identified using BLAST searches of UniProt database^6^. The multiple sequence alignment was performed using ClustalW at the European Bioinformatics Institute server. MEGA5 was used to construct the phylogenetic tree based on the neighbor-joining algorithm with default parameters. Bootstrap values were calculated from 1000 iterations. Protein structure modeling and prediction of PPR14 was completed using Phyre2 web portal (Kelley et al., 2015).

## Results

### PPR14 Is a Canonical P-Type PPR Protein Targeted to Mitochondria

**FIGURE 1:**
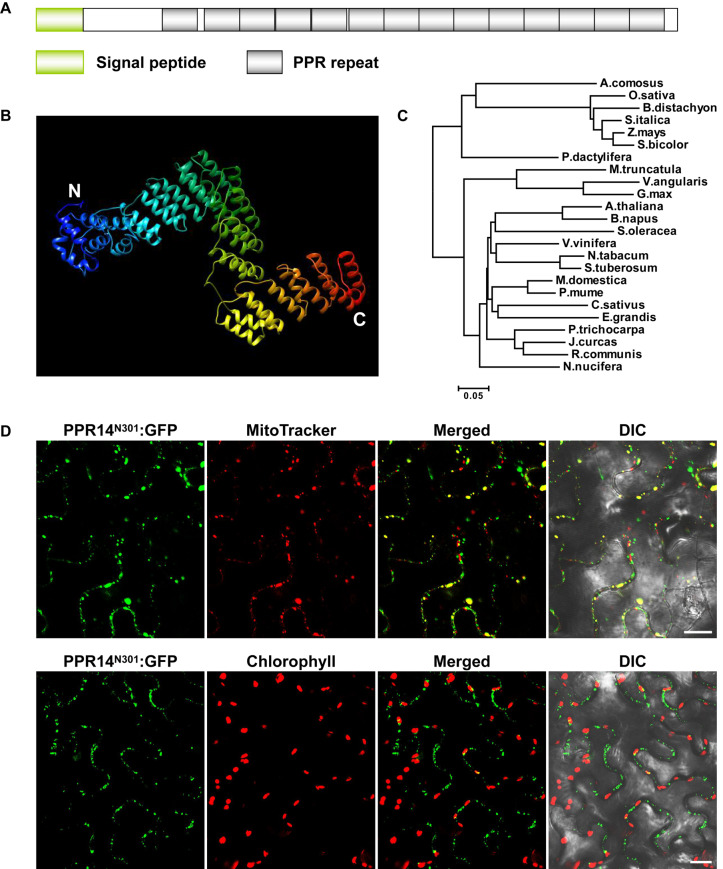
PPR14 is a P-type PPR protein localized in mitochondria. **(A)** The predicted PPR14 protein contains 14 PPR motifs. **(B)** Predicted structure of PPR14 protein. This model was predicted using Phyre2 (http://www.sbg.bio.ic.ac.uk/phyre2/html/page.cgi?id=index) based on template PPR10. Blue alpha helices indicate the N-terminus and red helices indicate the C-terminus of PPR14. **(C)** Phylogenetic analysis of PPR14 proteins in different species. **(D)** Subcellular localization of PPR14 protein. A PPR14^*N*301^-GFP fusion protein was transiently expressed in epidermal cells of *N*. *benthamiana* leaves. Mitochondria were stained by MitoTracker. Fluorescence signals from PPR14^*N*301^-GFP, MitoTracker, and chloroplast autofluorescence were detected by a confocal laser microscope. DIC, differential interference contrast; N301, the N terminus 301 amino acids of PPR14. Bars = 20 μm.

*PPR14* is an intronless gene that encodes a 640-aa protein containing 14 PPR motifs (Figure 1A). The protein does not have the C-terminal E, E+, and DYW domain (Lurin et al., 2004), indicating that PPR14 is a canonical P-type PPR protein. In recent years, the crystal structures of several PPR proteins have been revealed (Ban et al., 2013; Ke et al., 2013; Yin et al., 2013). We modeled the PPR14 protein based on the P-type PPR protein PPR10 (Yin et al., 2013) using Phyre2 (Kelley et al., 2015). The model showed that PPR14 has a series of paired antiparallel alpha helices forming a superhelix (Figure 1B). Phylogenetic analysis of PPR14 orthologs from 24 representative plant species which are classified as the lineages of monocots and eudicots shows that monocots and eudicots form two separate clades, and the maize PPR14 protein is conserved in monocots (Figure 1C).

Most PPR proteins are localized in mitochondria or plastids (Colcombet et al., 2013). The TargetP algorithm predicted PPR14 a mitochondrial localization^7^. To experimentally test, we fused the N-terminal 301 amino acids of PPR14 with the GFP in the pGWB5 vector, and transiently expressed the fusion protein in epidermal cells of tobacco leaves. GFP signals of PPR14^*N*301^-GFP were detected in small spots that overlapped with mitochondria stained with Mito Tracker Red (Figure 1D). We also detected the chlorophyll autofluorescence of chloroplasts, and the GFP signals did not merged with the chloroplasts which are much larger than the mitochondria. In addition, we fused the full-length PPR14 with the GFP in the pBI221 vector. The mitochondrial marker F1-ATPase-γ-RFP protein (Jin et al., 2003) and PPR14-GFP fusion protein were transiently co-expressed in Arabidopsis mesophyll protoplasts. Analysis of GFP and RFP signals by a confocal laser-scanning microscope showed that the GFP signals overlapped with the RFP signals (Supplementary Figure S1A). Whereas, free GFP as a negative control was found in cytoplasm (Supplementary Figure S1B). Together, these results indicate that PPR14 protein is localized in the mitochondrion.

### Phenotypic and Genetic Analysis of *ppr14-1*

**FIGURE 2:**
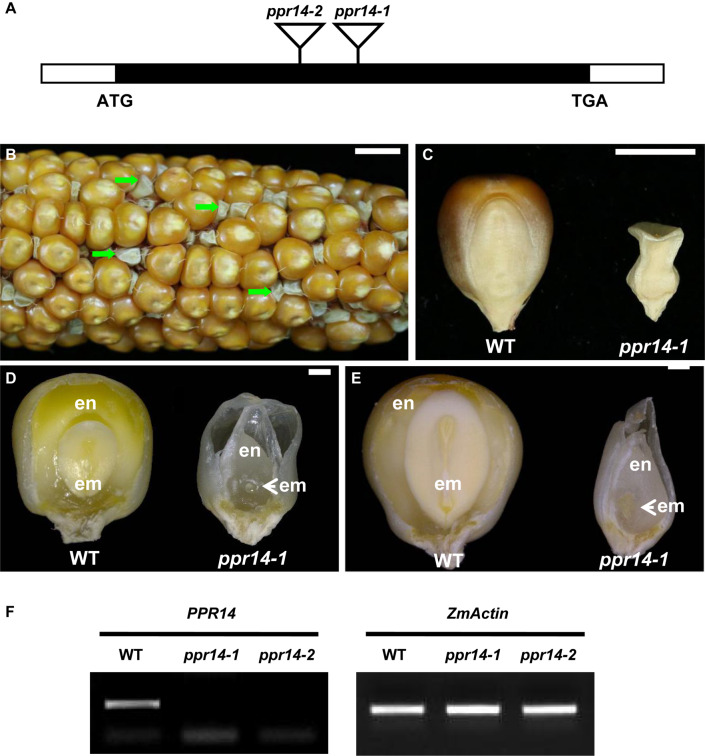
Phenotype of the *ppr14* mutant. **(A)** Gene structure of *PPR14* and locations of the *Mu* insertions in two independent alleles. The black box represents protein translated region, and white boxes represent the 5′ and 3′ untranslated regions. *Mu* insertion sites of *ppr14* alleles are indicated by triangles. **(B)** The selfed ear segregates 3:1 for wild-type and *ppr14-1* mutants (green arrows). **(C)** Comparison of mature wild-type (WT) and *ppr14-1* mutant kernel. **(D,E)** Dissection of WT and *ppr14* kernels at 13 **(D)** and 21 **(E)** days after pollination (DAP). en, endosperm; em, embryo. **(F)** RT-PCR analysis of *PPR14* expression in *ppr14* mutant alleles and WT seeds at 17 DAP. Bars = 1 cm in **(B)**, 5 mm in **(C)**, 1 mm in **(D,E)**.

Two Mutator insertional mutants were identified from the UniformMu mutant population (McCarty et al., 2005). The *ppr14-1* allele carries a *Mu* insertion at 988 bp, and the *ppr14-2* allele carries a *Mu* insertion at 751 bp downstream from the ATG in *PPR14*, respectively (Figure 2A). The selfed progenies of the *ppr14-1* heterozygotes segregated 3:1 for wild-type and *emp* kernels (Figure 2B), indicating the mutation is monogenic, recessive, and nuclear. Comparing with the wild-type, the *ppr14* mutant kernels were small, white, and wrinkled (Figures 2B,C). At 13 DAP, the wild-type kernel developed a normal embryo and a starchy endosperm, while the *ppr14* mutant embryos were arrested at the transition stage and the endosperms showed slow development (Figure 2D). At 21 DAP, the wild-type kernel developed an embryo with visible shoot apical meristem and a starch-filled endosperm, but the mutant kernel developed a tiny embryo and a watery endosperm (Figure 2E). These results indicate that the *ppr14* mutation severely arrests the embryo and endosperm development. As a result, the *ppr14-1* mutant is embryo lethal.

To test the linkage, co-segregation analysis was performed by using *PPR14*-specific and *Mu* TIR8 primers in a segregating population created by selfing a heterozygous *ppr14-1*/*PPR14* plant. Genomic DNA was extracted from 62 individual plants and no recombination was detected, indicating that the *Mu* insertion was tightly linked to the *ppr14-1* mutation (Supplementary Figure S2A). To confirm that the mutation in *PPR14* gene is responsible for the *emp* phenotype, we analyzed the additional *Mu* insertional line, *ppr14-2*. The selfed progeny of *ppr14-2* heterozygote and progeny from reciprocal crosses with *ppr14-1* heterozygotes all segregated *emp* kernels alike to *ppr14-1* (Supplementary Figure S2B). These results demonstrate that *PPR14* is the causative gene for the *emp* phenotype. We tested *PPR14* expression by RT-PCR in the 17 DAP kernels of *ppr14-1* and *ppr14-2* alleles. No wild-type *PPR14* transcript was detected in both alleles (Figure 2F), indicating that *ppr14-1* and *ppr14-2* are probably null mutations.

To gain information on *PPR14* expression pattern, we performed qRT-PCR on major tissues and developing kernels. The results indicated that *PPR14* was expressed in all vegetative and reproductive tissues tested (Supplementary Figure S3). Expression was relatively higher in leaves and ears than that in roots and silk. During seed development, *PPR14* is expressed at a higher level in the early developing kernels at 5 DAP than at later stages. This suggests that *PPR14* is likely to be expressed ubiquitously in all plant tissues, and throughout plant growth and development.

### Embryo and Endosperm Development Is Arrested in *ppr14-1*

**FIGURE 3:**
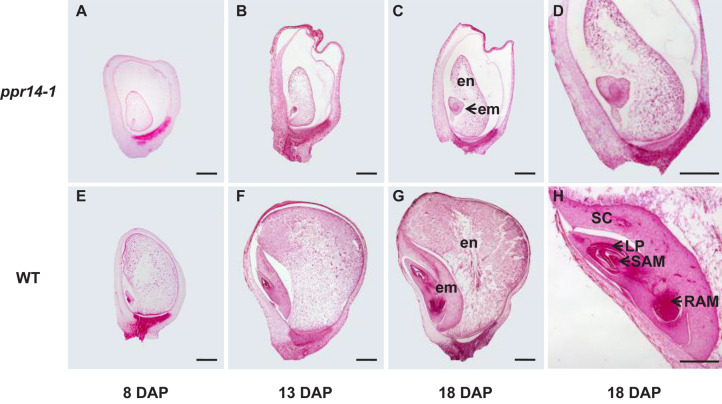
*PPR14* mutation arrests embryo and endosperm development. **(A–H)** Developmental analysis of wild-type (WT; **E–H**) and *ppr14* mutant **(A–D)** kernels at 8 **(A,E)**, 13 **(B,F)**, and 18 **(C,D,G,H)** days after pollination (DAP). Enlarged views of the *ppr14* mutant **(D)** and WT **(H)** embryos at 18 DAP. em, embryo; en, endosperm; LP, leaf primordia; RAM, root apical meristem; SAM, shoot apical meristem; SC, scutellum. Bars = 1 mm.

To determine the stage of the developmental arrest, we compared the seed development between homozygous *ppr14-1* and wild-type in a segregating ear by light microscopy. The *ppr14* kernels were visually different from the wild-type at 8 DAP. The mutant embryo stays at the transitional stage with an undifferentiated club-shaped structure (Figure 3A), while the wild-type embryo reaches the coleoptilar stage and starts to differentiate scutellum, coleoptile, and apical meristem (Figure 3E). At 13 DAP, the *ppr14* embryos remain at the transition stage, except for the embryo proper with the top densely packed with mitotic cells and forming a lower suspensor (Figure 3B). In contrast, the wild-type embryos reach late embryogenesis stage with visible scutellum, leaf primordia, shoot apical meristem, and root apical meristem (Figure 3F). At 18 DAP, the mutant embryos grow slightly, remaining as undifferentiated embryos and suspensor (Figures 3C,D), whereas the wild-type embryos develop three to four leaf primordia and a primary root (Figures 3G,H). The endosperm development is also delayed in *ppr14-1* mutant. The mutant endosperms are much smaller than the wild-type at all stages, which creates a large cavity between the pericarp and endosperm (Figures 3A–C,E–G). These results suggest that the embryo development of *ppr14* mutant was blocked at the transition stage and the endosperm development was also severely delayed.

### PPR14 Is Required for the Splicing of *nad2* Intron 3, *nad7* Introns 1 and 2 in Mitochondria

**FIGURE 4:**
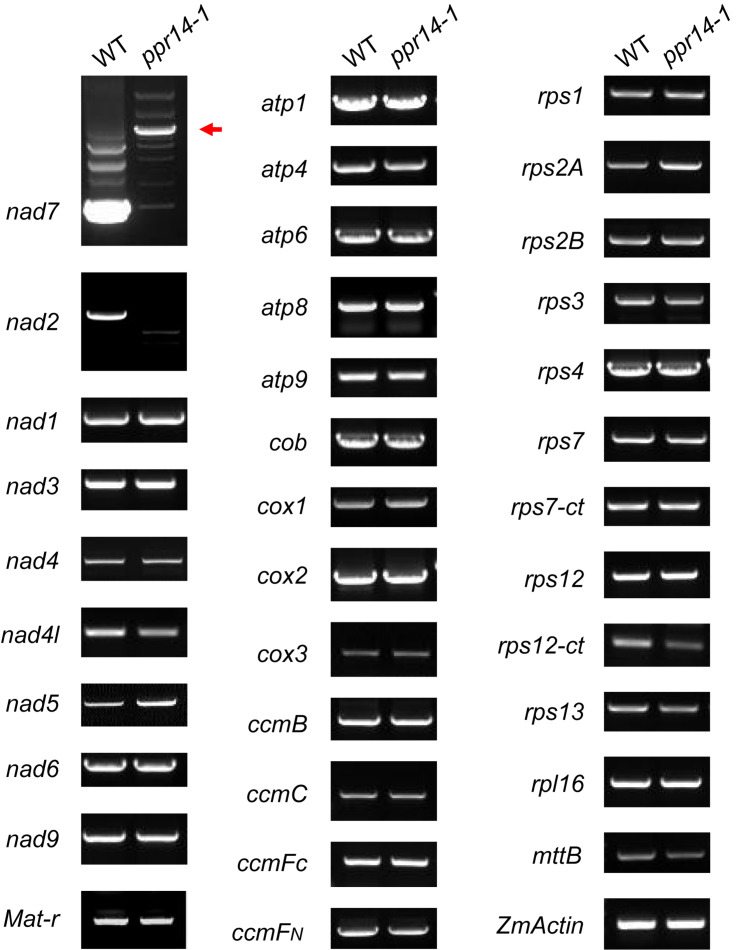
RT-PCR analysis of mitochondrial gene transcripts in the *ppr14-1* mutant and wild-type. The mature *nad2* and *nad7* transcripts were nearly abolished in the *ppr14-1* mutant. Red arrow indicates the large fragment of *nad7* in the *ppr14-1* mutant. *ZmActin* was used to normalize the quantifications.

P-type PPR proteins are involved in RNA cleavage, intron splicing, and translation in plant mitochondria (Barkan and Small, 2014). To determine the function of PPR14, we then examined the transcript levels of the 35 mitochondrial protein-coding genes in the wild-type and *ppr14-1* mutants. Total RNA was extracted from the 16-DAP kernels of *ppr14-1* mutant and wild-type from a segregating ear. The pericarp was removed carefully to prevent contamination of maternal tissues. RNA samples were treated with RNase-free DNase I to eliminate DNA contamination. 35 pairs of primers of mitochondrial protein-coding genes were used to amplify the transcripts (Liu et al., 2013). *ZmActin* (GRMZM2G126010) was used as the normalization control. RT-PCR analysis revealed that mature *nad2* and *nad7* transcripts were nearly abolished in the *ppr14-1* mutants (Figure 4). The remaining 33 mitochondrial genes showed no significant differences between the *ppr14-1* mutant and wild-type. In the *ppr14-1* mutant, a large fragment (red arrow) was produced and sequencing revealed that this fragment contains introns 1 and 2 of the *nad7* transcript. This result indicates that PPR14 may be required for the splicing of *nad7* introns 1 and 2.

**FIGURE 5:**
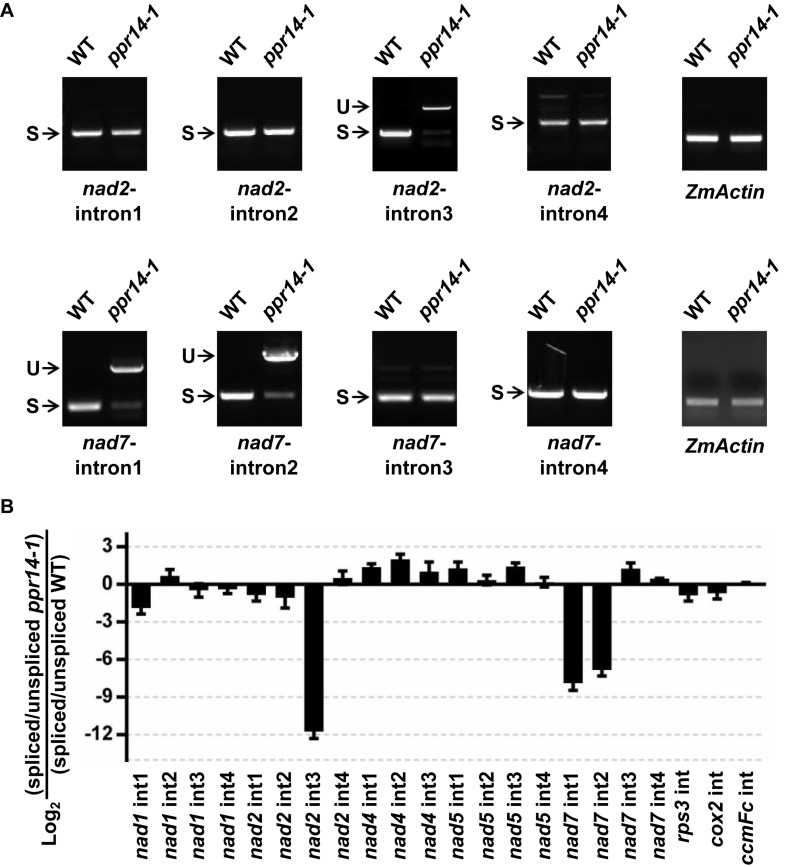
PPR14 is involved in the splicing of *nad2* intron 3 and *nad7* introns 1 and 2. **(A)** Detailed RT-PCR analysis of *nad2* and *nad7* splicing. *ZmActin* was used to normalize the quantifications. S, intron spliced; U, intron unspliced. **(B)** QRT-PCR analysis of 22 introns of mitochondrial gene transcripts in the *ppr14-1* mutant and wild-type (WT). RNA was extracted from *ppr14-1* mutant and WT kernels of three independent ears. *ZmEF1*α was used to normalize the quantifications. Values and error bars represent the mean and standard deviation of three biological replicates, respectively.

The maize *nad2* gene contains five exons, which are present in two different regions of the mitochondrial genome. The formation of mature *nad2* transcript requires one *trans*-splicing and three *cis*-splicing events (Supplementary Figure S4A). To test whether the loss of mature *nad2* is caused by intron-splicing deficiency, we analyzed the splicing efficiency of each intron by RT-PCR using four pairs of primers positioned in the adjacent intron-flanking exons. The results showed that the splicing of intron 3 is nearly abolished in the *ppr14-1* mutant (Figure 5A). In contrast, the splicing of other introns are normal. This splicing deficiency is also found in the *ppr14-2* allele (Supplementary Figure S4A). The mitochondrial *nad7* gene contains four *cis*-splicing introns (Supplementary Figure S4B). To validate whether splicing deficiency of *nad7* introns 1 and 2 is responsible for the large fragment of *nad7* transcript in *ppr14-1* mutant, we analyzed the splicing efficiency of each intron by the method similar to *nad2*. As shown in Figure 5A, the splicing of *nad7* introns 1 and 2 is dramatically reduced in the *ppr14-1* mutant, while the other introns are spliced normally. In the images of *nad7*-intron1 and *nad7*-intron2, the weak bands at “S” position in *ppr14-1* indicate that very few introns can be spliced normally. As a result, large bands containing unspliced introns accumulated in the *ppr14-1* mutant. This result is also confirmed in the *ppr14-2* allele (Supplementary Figure S4B). Therefore, PPR14 is required for splicing of *nad2* intron 3 and *nad7* introns 1 and 2.

To corroborate the results of the RT-PCR analysis, we carried out quantitative RT-PCR (qRT-PCR) analysis to examine all group II intron-splicing efficiency in mitochondria (Figure 5B). Total RNA was extracted from *ppr14-1* mutant and wild-type of three independent ears at 11 DAP. The result is consistent with that of RT-PCR, suggesting that *PPR14* is required for the splicing of *nad2* intron 3 and *nad7* introns 1 and 2. We tried to analyze the potential binding sites of PPR14 based on the 6,1′-codes proposed by Barkan et al. (2012) and the conserved sequences of these three introns. Unfortunately, we failed to obtain positive sequences for recognition.

### Deficiency of the *nad2* and *nad7* Intron Splicing Affects Mitochondrial Complex I Assembly

**FIGURE 6:**
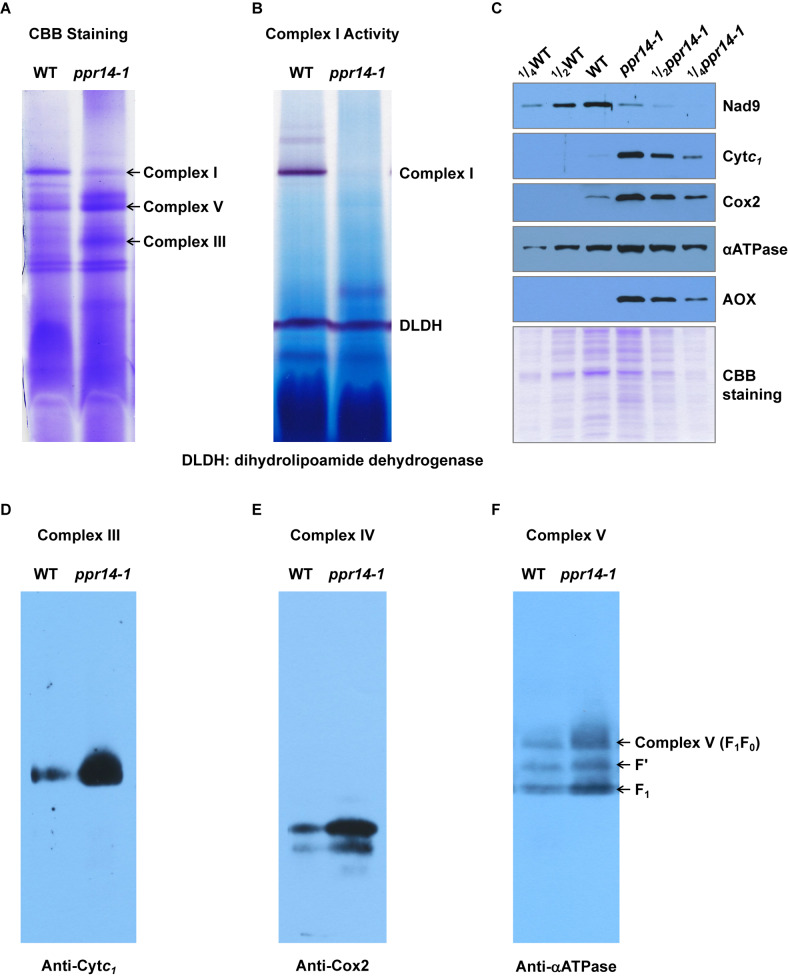
*PPR14* mutation dramatically reduces complex I assembly and activity. **(A)** Blue native (BN)-PAGE analysis of mitochondrial complexes. The BN gel was stained with Coomassie Brilliant Blue (CBB). **(B)** NADH dehydrogenase activity of mitochondrial complex I. DLDH is used as a loading control. **(C)** Immunodetection of *ppr14-1* mutant and wild-type (WT) mitochondrial proteins. **(D–F)** Accumulation of mitochondrial complex III, IV, and V in *ppr14-1* mutant and wild type (WT). The BN gels were transferred onto the PVDF membranes and probed with specific antibodies against Cyt*c*_1_ **(D)**, Cox2 **(E)**, and αATPase **(F)**.

In plant mitochondria, Nad2 is a core subunit of the membrane arm in the L-shaped complex I, and Nad7 is a core subunit of the peripheral arm (Subrahmanian et al., 2016). In the *ppr14* mutants, the lack of mature *nad2* and *nad7* transcripts will lead to the absence of Nad2 and Nad7 proteins. To investigate the effect of Nad2 and Nad7 deficiency on complex I assembly and its NADH dehydrogenase activity, we performed BN-PAGE and complex-I activity assay. Crude mitochondria were isolated from embryo and endosperm of *ppr14-1* mutants and wild-type siblings and were analyzed by BN-PAGE. The result revealed that complex I was dramatically reduced in the *ppr14-1* mutants compared with the wild-type siblings, whereas complex III and complex V were remarkably increased (Figure 6A). By using the activity of dihydrolipoamide dehydrogenase (DLDH) as a loading control, the activity of complex I was lost in the *ppr14-1* mutant (Figure 6B).

To investigate whether the lack of Nad2 and Nad7 affects components of other complexes, mitochondrial proteins were separated on SDS-PAGE and subjected to western blotting analysis with specific antibodies against Nad9 (complex I), Cyt*c*_1_ (complex III), Cox2 (complex IV), and αATPase (complex V). The Coomassie Brilliant Blue (CBB) staining was used as a loading control. The results indicate that the lack of Nad2 and Nad7 reduced Nad9 protein abundance dramatically, while the levels of Cyt*c*_1_, Cox2, and αATPase were remarkably increased in the *ppr14-1* mutants (Figure 6C). To further examine the assembly and amount of complex III, IV, and V in *ppr14-1* mutant, the BN gels were transferred onto PVDF membranes and probed with antibodies against Cyt*c*_1_ (Figure 6D), Cox2 (Figure 6E), and αATPase (Figure 6F). The results showed that these proteins as in the position of complex III, IV, and V accumulated substantially in *ppr14-1* mutant. This result is consistent with the CBB staining (Figure 6A) and western blotting (Figure 6C). Similar increases in these complexes are also reported with other complex I mutants such as *nmat1*, *4*, and *mtl1* mutants in Arabidopsis (Cohen et al., 2014; Haïli et al., 2016) and *emp8* and *emp12* mutants in maize (Sun et al., 2018, 2019).

The damage of mitochondrial respiratory chain may lead to an increase in the alternative respiratory pathway characterized by alternative oxidase (AOX). Indeed, AOX protein was strongly induced in the *ppr14-1* mutant (Figure 6C). Both RT-PCR and qRT-PCR analyses showed that both *AOX2* and *AOX3* transcripts were substantially increased in the *ppr14-1* mutant compared with the wild-type (Supplementary Figures S5A,B). QRT-PCR analyses showed that the level of *AOX2* transcripts is increased about 32 times and *AOX3* by about 8 times in the *ppr14-1* mutant compared with the wild-type (Supplementary Figure S5B). Together, these results indicate that the splicing defects in *nad2* intron 3 and *nad7* introns 1 and 2 result in the absence of Nad2 and Nad7 proteins, which disrupts complex I assembly and abolishes its activity in the respiratory chain, which possibly, in turn, causes the accumulation of other complexes and up-regulation of the alternative respiratory pathway.

### PPR14 Interacts With PPR-SMR1 and Zm-mCSF1

Emerging evidence suggests several splicing factors reside in high molecular weight complexes in chloroplasts, which include CRM, PPR, APO, and PORR proteins (Asakura et al., 2008; Hammani and Barkan, 2014; Reifschneider et al., 2016). Our results showed that the inefficient splicing of *nad2* intron 3 and *nad7* intron 2 was found in both *ppr14* and *ppr-smr1* (Chen et al., 2019), and the inefficient splicing of *nad2* intron 3 in *ppr14* and *Zm-mcsf1* (Chen et al., 2019). Additionally, the splicing efficiency of *nad2* intron 3 was reduced in *ppr14*, *ppr-smr1*, and *Zm-mcsf1*. Together, these results raise a strong possibility that these two PPR proteins and Zm-mCSF1 may interact physically to mediate the intron splicing in mitochondria.

**FIGURE 7:**
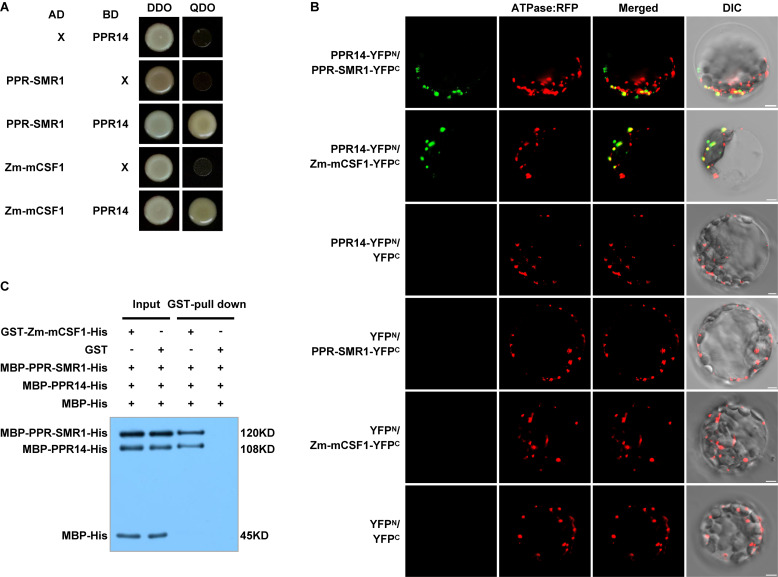
PPR14 protein interacts with PPR-SMR1 and Zm-mCSF1. **(A)** Yeast two-hybrid (Y2H) analysis of PPR14 and PPR-SMR1 interaction, PPR14 and Zm-mCSF1 interaction. The Y2HGold strain harboring the indicated bait and prey constructs were spotted on synthetic dropout (SD)/-Leu-Trp (without Leu and Trp; DDO) and SD/-Ade-Leu-Trp-His (without Ade, Leu, Trp, and His; QDO). Yeast cultures on DDO control plates prove the existence of both plasmids. Positive interactions were verified by growth on QDO plates. **(B)** *In vivo* interactions between PPR14, PPR-SMR1, and Zm-mCSF1 proteins examined by BiFC. Yellow fluorescent protein (YFP) is split into N-terminus (YFP^*N*^) and C-terminus (YFP^*C*^). PPR14 is fused with YFP^*N*^, PPR-SMR1 and Zm-mCSF1 are fused with YFP^*C*^, respectively. The indicated combinations of -YFP^*N*^ and -YFP^*C*^ fusion proteins were transiently co-expressed in protoplasts of Arabidopsis leaves. Mitochondria were labeled by F1-ATPase-γ-RFP marker. Non-targeted YFP^*N*^ and YFP^*C*^ were used as negative controls. YFP signals and RFP signals were detected by a confocal laser microscope. Bars = 5 μm. **(C)** Pull-down assay for interactions between PPR14, PPR-SMR1, and Zm-mCSF1 proteins. Equal amounts of MBP-PPR14-His, MBP-PPR-SMR1-His, and MBP-His were combined with GST beads pre-incubated with GST-Zm-mCSF1-His or GST (input). Both input samples and pulled-down samples were analyzed by immunoblot with anti-MBP antibody. “ + ” and “−” indicate the presence and absence of corresponding proteins in the reactions, respectively.

To explore this possibility, we first tested these proteins for physical interactions by yeast two-hybrid (Y2H) analyses. In our Y2H assays, predicted mature PPR14 (PPR14_39__–__640_) can interact with PPR-SMR1 (PPR-SMR1_49__–__787_) and Zm-mCSF1 (Zm-mCSF1_30__–__424_) (Figure 7A). Also, PPR-SMR1 interacts with Zm-mCSF1 (Chen et al., 2019). To confirm the interactions of PPR14 with PPR-SMR1 and Zm-mCSF1 *in vivo*, we performed bimolecular fluorescence complementation (BiFC) assay in Arabidopsis mesophyll protoplasts. YFP was split into YFP^*N*^ and YFP^*C*^, and PPR14 was fused with YFP^*N*^, PPR-SMR1 and Zm-mCSF1 with YFP^*C*^. The YFP fluorescence can be detected in the combination of PPR14-YFP^*N*^ and PPR-SMR1-YFP^*C*^ or the combination of PPR14-YFP^*N*^ and Zm-mCSF1-YFP^*C*^, indicating that PPR14 interacts with PPR-SMR1 and Zm-mCSF1 physically *in vivo*. As negative controls, the combinations of PPR14-YFP^*N*^ and YFP^*C*^, YFP^*N*^ and PPR-SMR1-YFP^*C*^, YFP^*N*^ and Zm-mCSF1-YFP^*C*^, YFP^*N*^ and YFP^*C*^ were co-expressed in Arabidopsis protoplasts, but no YFP fluorescence can be detected (Figure 7B). To exclude the possibility that these three proteins are sticky proteins, we examined their interactions with other proteins. In maize, the P-type PPR protein EMP8 is required for the splicing of three mitochondrial introns (Sun et al., 2018), and PPR20 is required for the splicing of *nad2* intron 3 (Yang et al., 2019). As a result, PPR14 could not interact with either EMP8 or PPR20 in Y2H analyses (Supplementary Figure S6) and BiFC assays (Supplementary Figure S7). In addition, PPR14 could not interact with another predicted PPR protein PPR-I (GRMZM2G089959) in Y2H system (Supplementary Figure S6). We also performed Y2H assays using PPR protein PPR-II (Zm00014a038761), CRM protein Zm-mCSF2 (GRMZM2G129615; homolog of Zm-mCSF1), and PORR protein EMP6 (Chettoor et al., 2015) with PPR-SMR1 and Zm-mCSF1. The results showed that PPR-SMR1 and Zm-mCSF1 could not interact with PPR-II, Zm-mCSF2, or EMP6 (Supplementary Figure S8). Taken together, we rule out the possibility that PPR14, PPR-SMR1, and Zm-mCSF1 are sticky proteins. Their interaction is specific. To corroborate the interaction between the three proteins, we performed a glutathione S-transferase (GST) pull-down assay with recombinant GST-Zm-mCSF1-His (6xHistidine), MBP-PPR-SMR1-His, MBP-PPR14-His, and MBP-His (Figure 7C). The MBP-His protein used as a negative control was added to the binding reaction. After incubation and washing, the GST beads combined with GST-Zm-mCSF1-His protein pulled down MBP-PPR-SMR1-His and MBP-PPR14-His, but did not enrich MBP-His. As a control, the GST beads combined with GST protein did not pull down MBP-PPR-SMR1-His, MBP-PPR14-His, and MBP-His. These results suggested that PPR14, PPR-SMR1, and Zm-mCSF1 may form a protein complex.

### Identification of the Interacting Domains in the PPR14/PPR-SMR1/Zm-mCSF1 Protein Interaction

**FIGURE 8:**
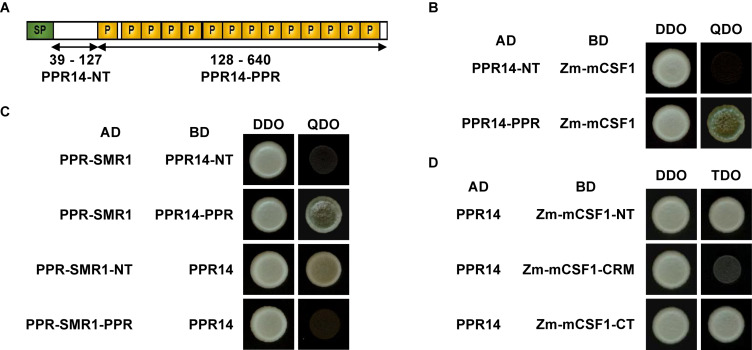
Protein fragments required for interactions of PPR14-PPR-SMR1-Zm-mCSF1. **(A)** Schematic diagram of full-length and split fragment fusions of PPR14 to GAL4 activating domain (AD) or GAL4 DNA binding domain (BD), respectively. PPR14 protein was split into two fragments: PPR14-NT (N-terminus, 39–127) and PPR14-PPR (128–640). **(B)** PPR14-PPR^*AD*^ physically interacts with Zm-mCSF1^*BD*^. **(C)** PPR-SMR1^*AD*^ physically interacts with PPR14-PPR^*BD*^. PPR-SMR1-NT^*AD*^ physically interacts with PPR14^*BD*^. **(D)** PPR14 interacts with the N terminus and C terminus of Zm-mCSF1.

To investigate the precise interaction regions between PPR14, PPR-SMR1, and Zm-mCSF1, PPR14 protein was split into two fragments: PPR14-NT (N-terminus, amino acids 39 to 127) and PPR14-PPR (amino acids 128 to 640) (Figure 8A). In addition, a series of proportional *PPR-SMR1* and *Zm-mCSF1* cDNAs have been cloned into the prey and bait constructs (Chen et al., 2019). Our results showed that PPR14-PPR domain not only interacts with mature PPR-SMR1 but also interacts with Zm-mCSF1 (Figures 8B,C). In turn, PPR-SMR1-NT (amino acids 49 to 193) and Zm-mCSF1-NT (amino acids 30 to 161) and CT (amino acids 371 to 424) are able to interact with PPR14, respectively (Figures 8C,D).

## Discussion

### The Splicing Defects in *nad2* and *nad7* Cause Mitochondrion Dysfunction and Defective Seed Development in the *ppr14* Mutants

Analysis of the mitochondrial transcript levels revealed that the splicing of *nad2* intron 3 is nearly abolished and the splicing of *nad7* introns 1 and 2 is dramatically reduced in the *ppr14* mutants (Figure 5 and Supplementary Figure S4). The failed splicing of these introns will undoubtedly cause a deficiency of these proteins. Both Nad2 and Nad7 are the core subunits of the L-shaped complex I in plant mitochondria. Nad2 is located in the membrane arm of the complex I, while Nad7 is located in the peripheral arm (Subrahmanian et al., 2016). In *ppr14-1* mutants, the lack of functional Nad2 and Nad7 proteins disrupts complex I assembly, abolishes complex I dehydrogenase activity, and reduces respiration rate (Figures 6A,B and Supplementary Figure S5C). Impairment of complex I assembly possibly, in turn, causes the dramatic increases of other complexes and induces the expression of AOX proteins, which up-regulates the alternative respiratory pathway in mitochondria (Figure 6C). A similar finding was also described in the *nMat1*, *nMat2*, *nMat4* (Keren et al., 2009, 2012; Cohen et al., 2014), and *emp16* mutants (Xiu et al., 2016), where splicing deficiency causes defect in the mitochondrial electron transfer chain.

Mitochondrial Complex-I is the major entry site of electrons from NADH oxidation into the electron transport chain, which is crucial for cellular metabolism in plants and many other organisms. Complex I dysfunction often causes retarded growth or defective seed development. In maize, splicing deficiency of *nad2* intron 4 in *emp16* mutants severely decreases assembly of complex-I and causes defective seed development (Xiu et al., 2016). In *smk1* mutants, the loss of C→U editing at the *nad7*-836 site in mitochondria dramatically reduces complex-I assembly and its dehydrogenase activity, and arrests both the embryo and endosperm development (Li et al., 2014). In Arabidopsis, the defective splicing of *nad2* intron 3 results in a deficiency in complex-I activity and disturbs both vegetative and reproductive development in *mterf15* mutants (Hsu et al., 2014). The lack of splicing in *nad7* intron 2 significantly reduces mitochondrial complex-I activity and leads to late germination, retarded growth, and delayed development phenotypes in *slo3* mutants (Hsieh et al., 2015). In *bir6* mutants, the reduced splicing of *nad7* intron 1 also disrupts assembly of mitochondrial complex I and causes slow growth phenotype (Koprivova et al., 2010). In addition, complex I dysfunction can cause antenatal cardiomyopathy, lethal infantile mitochondrial disease or adult onset neurodegenerative disorders in humans (Mimaki et al., 2012).

### PPR14, PPR-SMR1, and Zm-mCSF1 May Interact to Mediate Specific Intron Splicing

Organellar group II introns in higher plants have lost their autocatalytic activity and require various protein cofactors to facilitate the splicing *in vivo* (Brown et al., 2014). Although several PPR proteins (de Longevialle et al., 2007; Koprivova et al., 2010; des Francs-Small et al., 2014; Xiu et al., 2016; Chen et al., 2017), CRM domain-containing proteins (Till et al., 2001; Ostheimer et al., 2003; Zmudjak et al., 2013), and others are found to be required for intron splicing, it is not clear how these proteins work, together or independently. We revealed the function of PPR14 in the splicing of *nad2* intron 3 and *nad7* introns 1 and 2 in mitochondria. Similarly, the splicing of *nad2* intron 3 also requires PPR-SMR1 and Zm-mCSF1, and the phenotype of *ppr14* mutants is quite similar to the *ppr-smr1* and *Zm-mcsf1* mutants (Chen et al., 2019). Yeast two-hybrid analysis showed that PPR14 can bind to PPR-SMR1 and Zm-mCSF1 (Figure 7A). Moreover, PPR-SMR1 was found to interact with Zm-mCSF1 (Chen et al., 2019). The *in vivo* BiFC assay and *in vitro* pull-down assay confirm the interactions between the three proteins (Figures 7B,C). We hence propose that the splicing of *nad2* intron 3 required at least these three proteins and in a protein complex form. This finding is supported by the previous reports that several chloroplast splicing factors have been found in large ribonucleoprotein complexes in maize chloroplasts (Jenkins and Barkan, 2001; Till et al., 2001; Asakura and Barkan, 2007; Watkins et al., 2007; Asakura et al., 2008; Kroeger et al., 2009; Khrouchtchova et al., 2012). For example, THA8 was reported to promote the splicing of *trnA* intron in conjunction with the nucleus-encoded splicing factors RNC1 and WTF1 in the chloroplast stroma (Khrouchtchova et al., 2012). Similarly, a ribonucleoprotein complex containing at least five nucleus-encoded splicing factors has been reported to promote the *trans*-splicing of *psaA* intron 1 in *Chlamydomonas reinhardtii* chloroplast (Rivier et al., 2001; Merendino et al., 2006; Jacobs et al., 2013). While these proteins are all RNA-binding proteins, they could bind to the intron independently. Our study indicates that PPR14, PPR-SMR1, and Zm-mCSF1 may physically interact with each other in facilitating intron splicing in mitochondria. As existing evidence supports that the mitochondria and chloroplasts may share a conserved intron splicing mechanism, we speculate that intron splicing in chloroplasts may employ a similar machinery.

Since the removal of group II introns from exons requires the formation of rigid and conserved ribozyme-like tertiary structures within introns, and the fact that plant organellar introns have become highly degenerated and lost some of the essential sequences for the tertiary interactions between different domains. This protein complex could be the hub that connects different RNA domains (DI to DVI) to form the accurate catalytic conformation so that two transesterification reactions can proceed sequentially. However, in organellar group II introns, factors of the splicing machinery are thought to recognize the RNA fragments localized to DI, DIII, and DIV, and guide the folding of these introns into the correct active conformation (Ostheimer et al., 2005; Keren et al., 2008). Presumably, PPR14, PPR-SMR1, and Zm-mCSF1 bind to different conserved regions of *nad2* intron 3 and participate in the formation or maintenance of the conserved tertiary structure of this intron by protein-protein interaction.

In maize, the NB mitochondrial genome contains 22 group II introns. This raises a few questions as (1) why PPR14 is not involved in splicing of other 19 mitochondrial group II introns; (2) in addition to *nad2* intron 3, whether the splicing of remaining 21 introns requires a protein complex. Current evidence indicates that PPR proteins can specifically bind their target RNA sequences (Nakamura et al., 2003; Williams-Carrier et al., 2008; Pfalz et al., 2009; Prikryl et al., 2011; Ke et al., 2013). Barkan et al. (2012) and Takenaka et al. (2013) described the coding rule of RNA recognition by PPR proteins. Accordingly, PPR14 is likely to recognize and bind to specific sequences within *nad2* intron 3 and *nad7* introns 1 and 2, while sequences of the other 19 introns cannot be recognized by PPR14. Considering the large number of PPR proteins, CRM-domain containing proteins, and other families of proteins that are involved in intron splicing, most introns are likely to require multiple proteins. Based on our finding in this study, some of these proteins function in a complex which is required to maintain the autocatalytic state of the intron while other proteins may function independently in binding to the intron. Different introns may have different combinations with different splicing factors, such as the PPR proteins, CRM domain proteins, PORR domain family, maturases, and mTERFs which are required for intron splicing (Till et al., 2001; Ostheimer et al., 2003; Keren et al., 2009, 2012; des Francs-Small et al., 2012, 2014; Zmudjak et al., 2013; Cohen et al., 2014; Hsu et al., 2014; Sultan et al., 2016; Xiu et al., 2016; Chen et al., 2017). As indicated, the splicing of *nad7* intron 2 requires at least PPR14 and PPR-SMR1 (Chen et al., 2019), and may require some other unknown proteins in the maize mitochondria. In addition, further evidence to prove the PPR14-PPR-SMR1-Zm-mCSF1 complex *in vivo* is needed, for example, structural revelation of these three proteins and RNA in a complex which may also reveals which protein defines the RNA binding specificity.

## Data Availability Statement

The datasets generated for this study are available on request to the corresponding author.

## Author Contributions

H-CW and B-CT designed the research. H-CW conducted most of the experiments. Y-ZY, FS, SD, and X-LL participated in the BN gel analysis. H-CW, CX, and B-CT analyzed the data. H-CW, ZC, and B-CT wrote the manuscript.

## Conflict of Interest

The authors declare that the research was conducted in the absence of any commercial or financial relationships that could be construed as a potential conflict of interest.

## Footnotes

1 http://www.qiagen.com/

2 https://www.neb.com/

3 http://www.transgen.com.cn/

4 https://www.lifescience.roche.com/

5 http://www.agrisera.com/

6 http://www.uniprot.org

7 http://www.cbs.dtu.dk/services/TargetP/
