# Supplementary material for: PPR14 Interacts With PPR-SMR1 and CRM Protein Zm-mCSF1 to Facilitate Mitochondrial Intron Splicing in Maize
Source: Front Plant Sci. 2020 Jun 12;11:814. doi: 10.3389/fpls.2020.00814 (PMC7304344; doi:10.3389/fpls.2020.00814)
Supplement: Supplementary file 1 [file Data_Sheet_1.PDF]

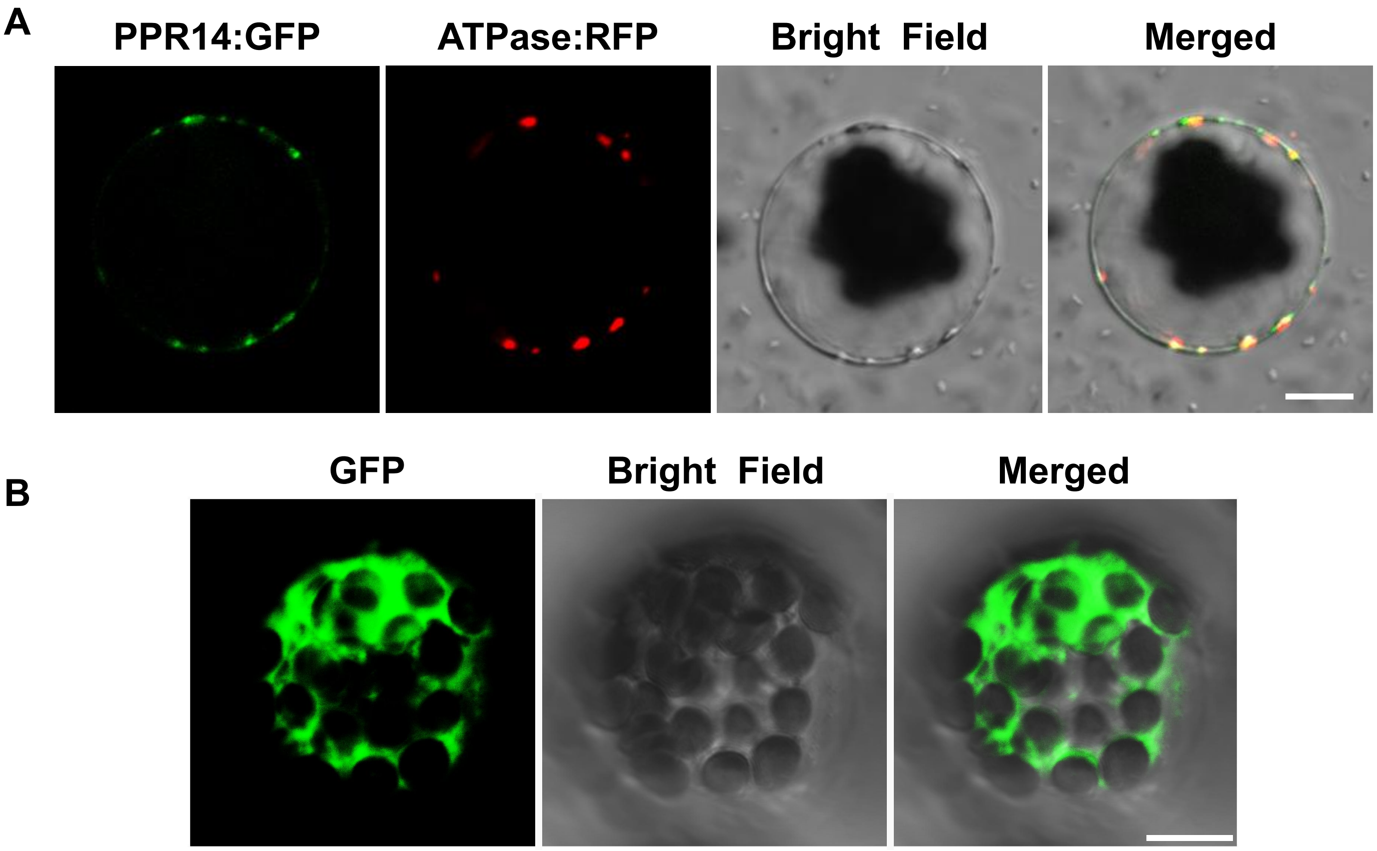

Fig. S1. Subcellular localization of PPR14 protein in Arabidopsis protoplasts. (A) Co-localization of GFP-tagged PPR14 and RFP-tagged mitochondrial marker protein F1-ATPase-γ. PPR14-GFP fusion protein and ATPase-RFP fusion protein were transiently co-expressed in protoplasts of Arabidopsis leaves. Fluorescence signals from PPR14-GFP (green) and ATPase-RFP (red) were detected by a confocal laser microscope. (B) Subcellular localization of free GFP in mesophyll protoplasts of Arabidopsis. Bars = 10 μm.

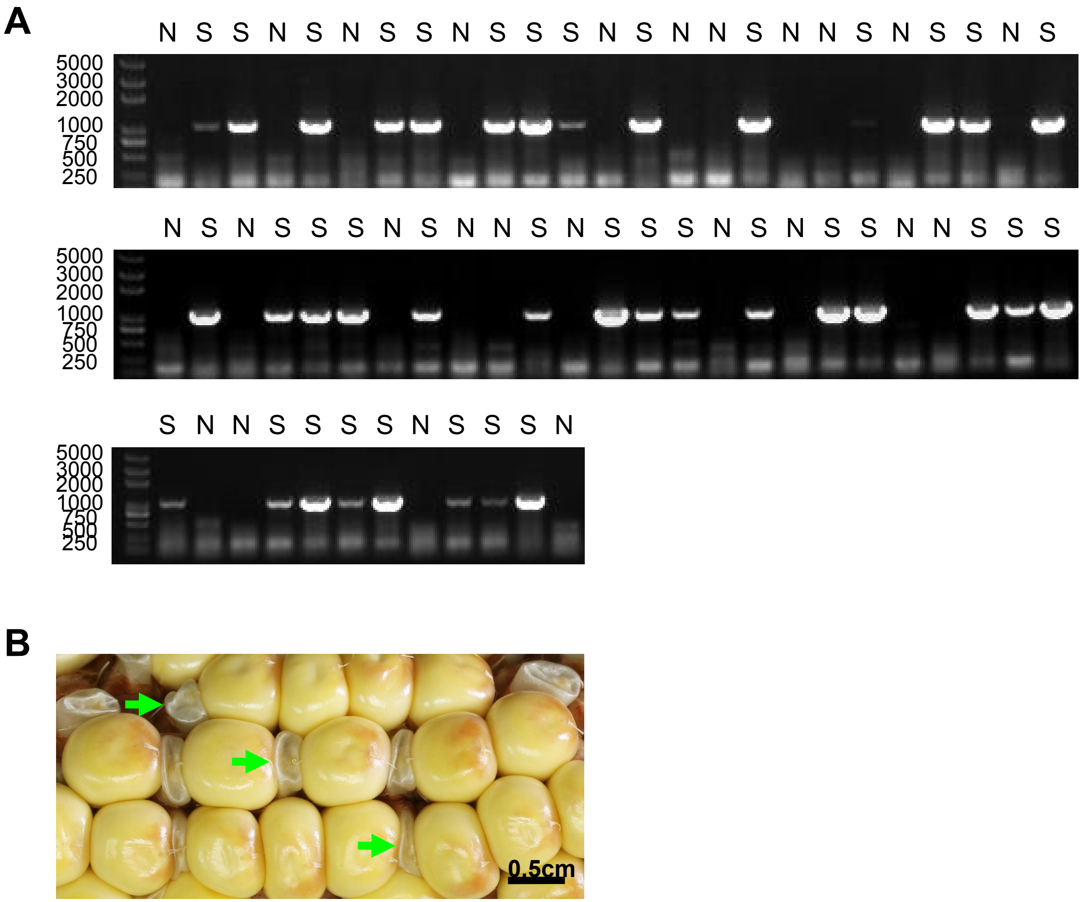

Fig. S2. *PPR14* linkage analysis. (A) Co-segregation analysis of a segregating population. 62 individual plants were genotyped by PCR amplification with 14-F2 and TIR8 primers at seedling stage. The PCR product at 1000 bp indicates the *Mu* insertion in *PPR14* gene. The phenotype of the individual selfed cobs was observed when they matured. N, nonsegregating (wild type); S, segregating (heterozygous *PPR14*/*ppr14*). (B) Cross betewen *ppr14-1* and *ppr14-2* produce ears segregating *empty pericarp* kernels (green arrows) at a 1:3 ratio (*emp*:WT).

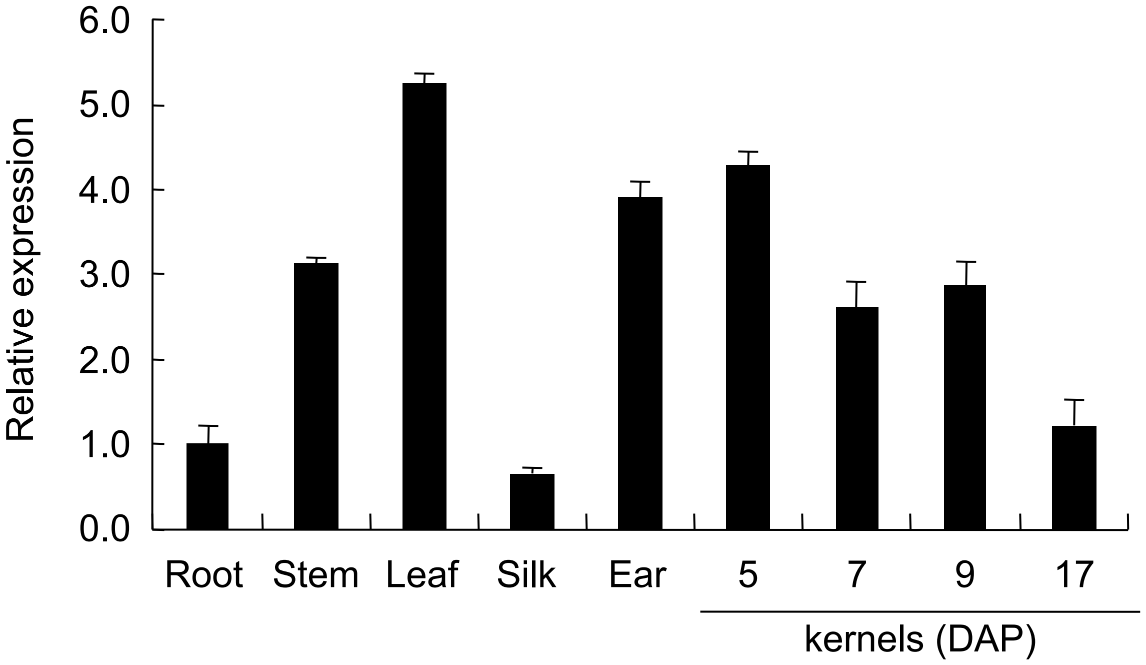

Fig. S3. *PPR14* expression in different tissues and developing kernels. *PPR14* expression in roots, stems, leaves, silk, ear, and kernels at different stages. *ZmEF1α* was used to normalize the quantifications. Values and error bars represent the mean and standard deviation of three biological replicates, respectively.

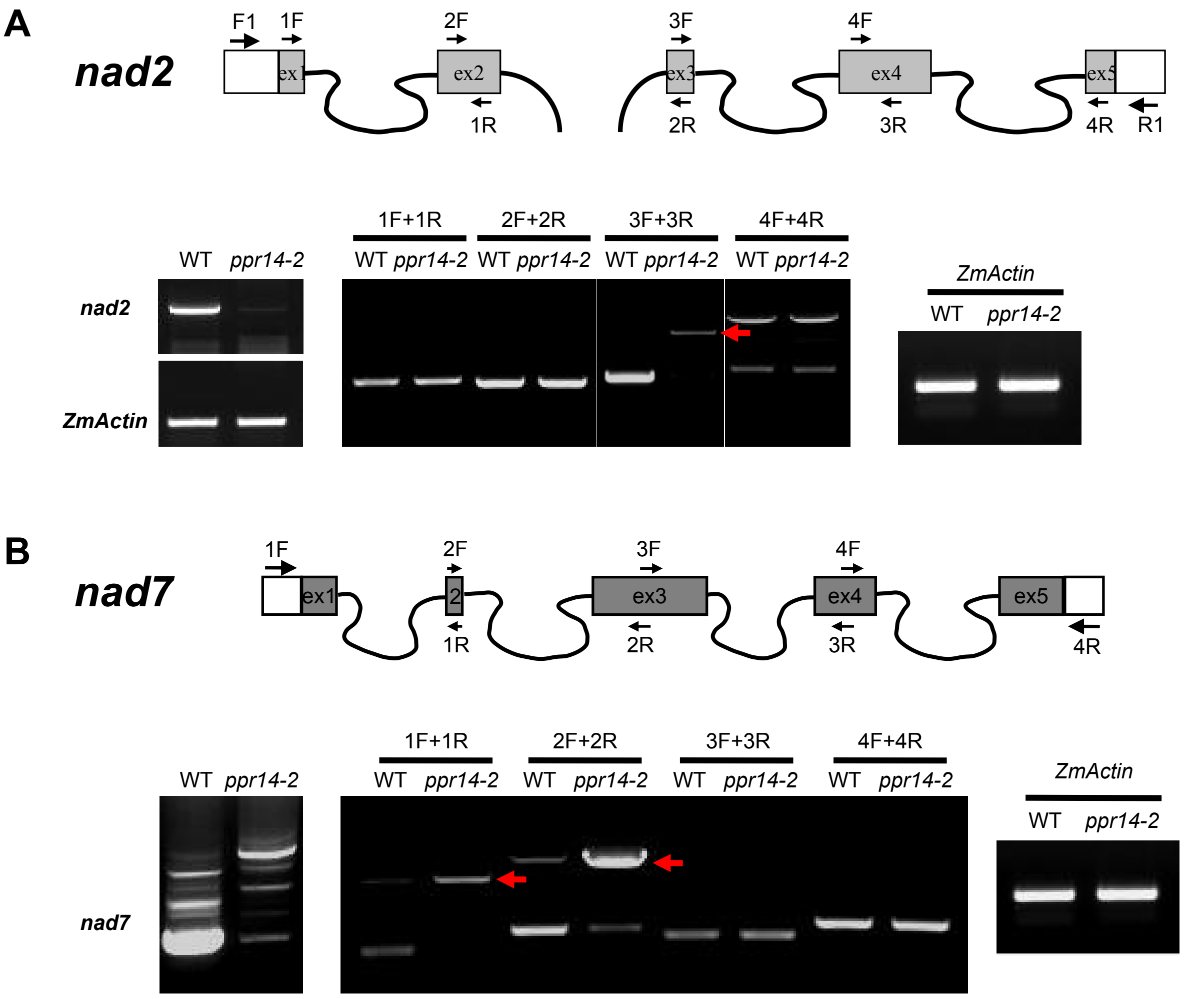

Fig. S4. Detailed analysis of *nad2* and *nad7* splicing in the *ppr14-2* mutant. (A) Gene structure and RT-PCR analysis of *nad2*. Exons are closed boxes and introns are lines. RT-PCR analysis of the *nad2* transcript in *ppr14-2* mutant and wild-type (WT) was performed using various combinations of the primers described above. Red arrow indicates the intron-containing products. The splicing of *nad2* intron 3 is nearly abolished in the *ppr14-2* mutant. (B) The *nad7* gene structure and RT-PCR analysis of the *nad7* transcript. The *nad7* gene has five exons that are joined by four *cis*-splicing events. Red arrows indicate the intron-containing products. *ppr14* mutation dramatically reduces splicing of *nad7* introns 1 and 2.

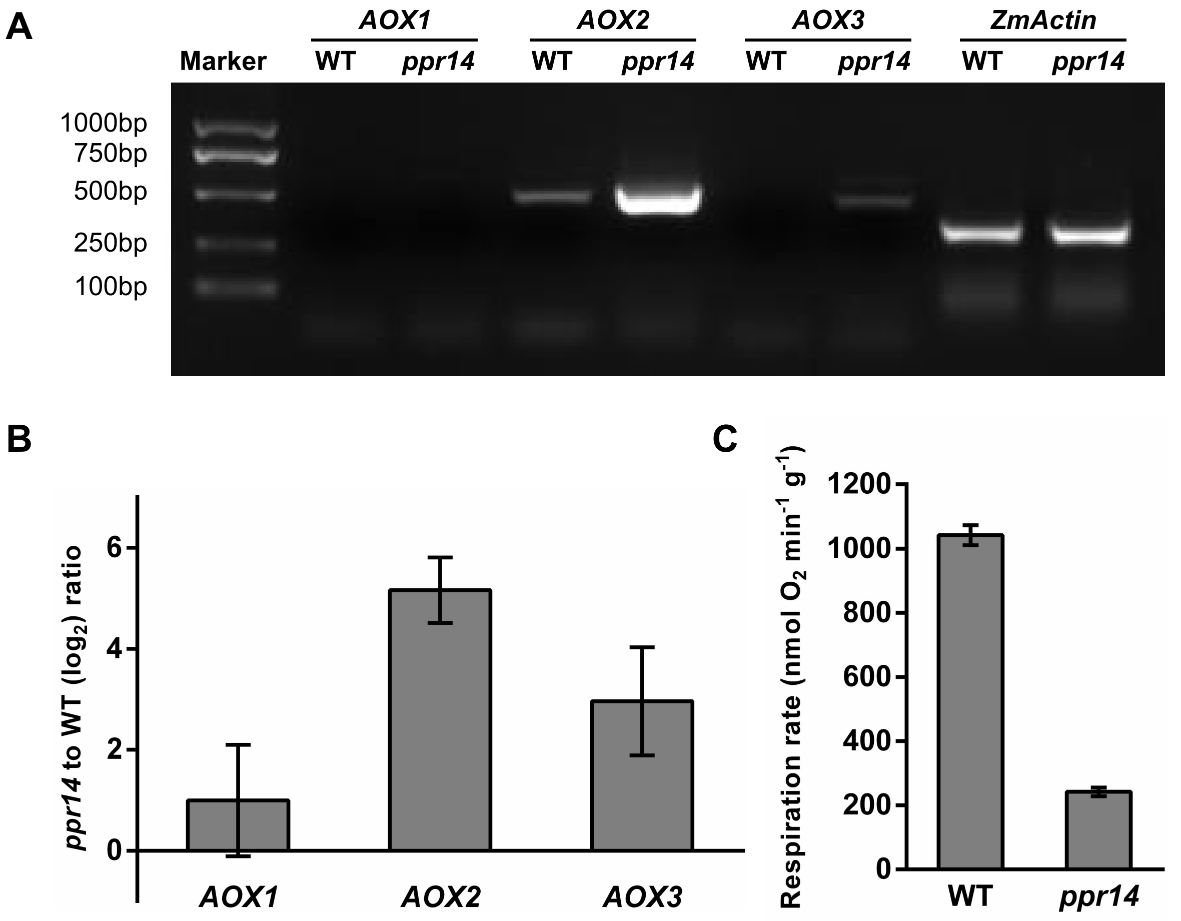

Fig. S5. *PPR14* mutation significantly increased *AOX2* gene expression and decreased respiration rate. (A) Semi-quantitative RT-PCR analysis of *AOX1*, *AOX2*, and *AOX3* gene expression in the wild-type (WT) and *ppr14-1* mutant kernels at 13 days after pollination (DAP). *ZmActin* was used to normalize the quantifications. (B) Real-time PCR analysis of *AOX1*, *AOX2*, and *AOX3* gene expression in the WT and *ppr14-1* mutant kernels at 13 DAP. *ZmEF1α* was used to normalize the quantifications. Values and error bars represent the mean and standard deviation of three biological replicates, respectively. (C) Respiration rate of wild-type (WT) and *ppr14-1* mutant kernels at 13 DAP.

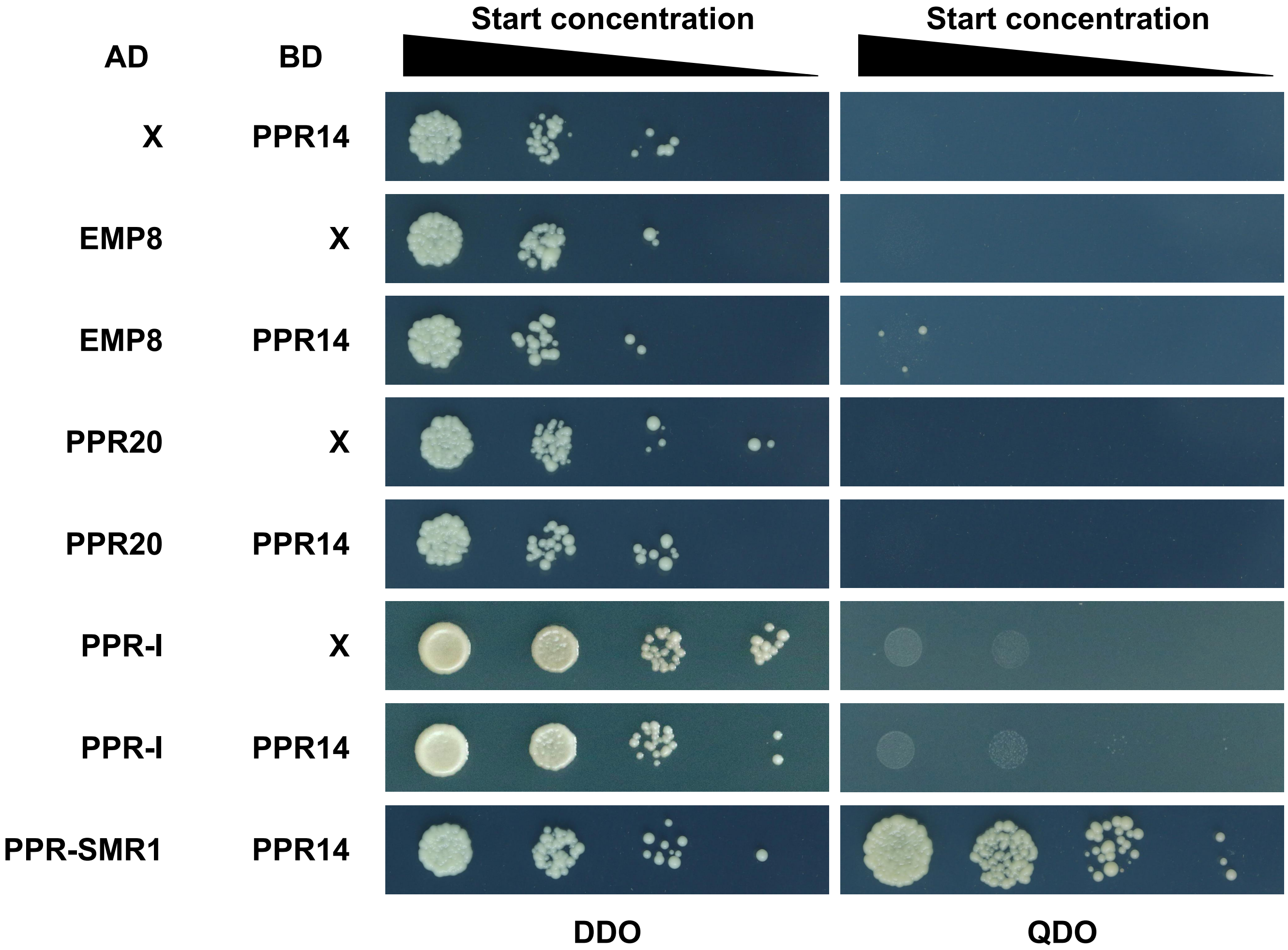

Fig. S6. PPR14 cannot interact with EMP8, PPR20, or PPR-I (GRMZM2G089959) in yeast two-hybrid system. The Y2HGold strain harboring the indicated bait and prey constructs were spotted on synthetic dropout (SD)/-Leu-Trp (without Leu and Trp; DDO) and SD/-Ade-Leu-Trp-His (without Ade, Leu, Trp, and His; QDO). Yeast cultures on DDO control plates prove the existence of both plasmids. Positive interactions were verified by growth on QDO plates.

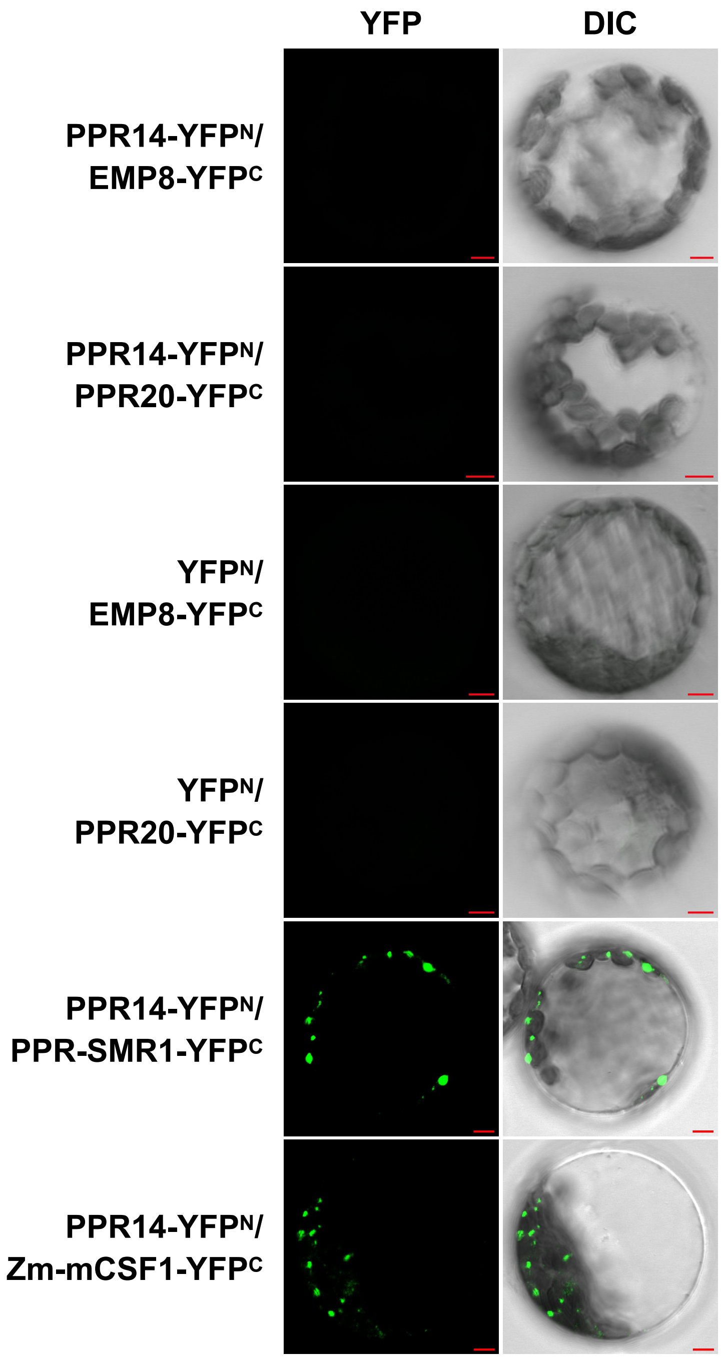

Fig. S7. PPR14 cannot interact with EMP8 or PPR20 in BiFC assay. Yellow fluorescent protein (YFP) is split into N-terminus (YFP^N) and C-terminus (YFP^C). PPR14 is fused with YFP^N, EMP8 and PPR20 are fused with YFP^C, respectively. The indicated combinations of -YFP^N and -YFP^C fusion proteins were transiently co-expressed in protoplasts of Arabidopsis leaves. Non-targeted YFP^N was used as negative control. PPR-SMR1 and Zm-mCSF1 were used as positive controls. YFP signals were detected by a confocal laser microscope. Bars = 5 μm.

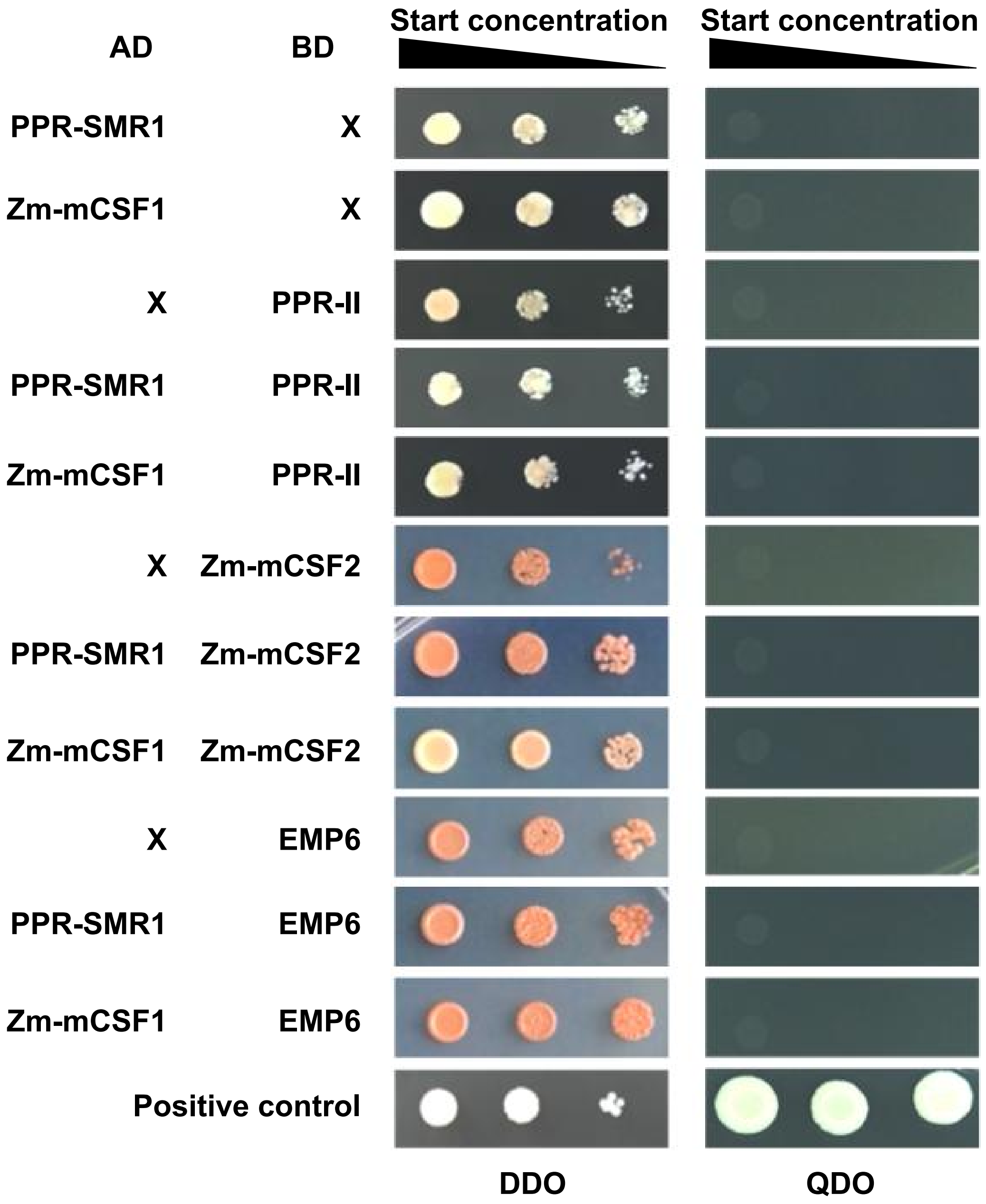

Fig. S8. PPR-SMR1 and Zm-mCSF1 cannot interact with PPR protein PPR-II (Zm00014a038761), CRM protein Zm-mCSF2 (GRMZM2G129615; homolog of Zm-mCSF1), or PORR protein EMP6 in yeast two-hybrid system. The Y2HGold strain harboring the indicated bait and prey constructs were spotted on synthetic dropout (SD)/-Leu-Trp (without Leu and Trp; DDO) and SD/-Ade-Leu-Trp-His (without Ade, Leu, Trp, and His; QDO). Yeast cultures on DDO control plates prove the existence of both plasmids. Positive interactions were verified by growth on QDO plates.

Table S1. Primers used in this study.

Primer name	Primer sequence (5' to 3')	Use
14-F1	CCGAGGAGCTGCTATGGG	PPR14 genotyping, RT-PCR
14-R1	CTCCAAATTGCCAAACCGGC	
14-F2	CACCATGCGTCGCTACTGCCAC	PPR14 genotyping
TIR8a	CGCCTCCATTTCGTCGAATCCCCTS	
TIR8b	CGCCTCCATTTCGTCGAATCCSCTT	
TIR8c	SGCCTCCATTTCGTCGAATCCCKT	
TIR8d	CGCCTCCATTTCGTCGAATCACCTC	
ZmActin-F1	TAGTTGAGAATGGCTGACGAGG	maize RNA normalization
ZmActin-R1	ATCTTCAGGCGAAACACGGAGC	
14-F3	CACCATGCGTCGCTACTGCC	Construct pENTR-PPR14^{N301} for localization
14-R2	CTCTTGGAACAGTGCTATGCATTG	
14-F4	ATTTGCGGCCGCCCCCTTCACC	Construct pENTR-PPR14 for localization
14-R3	TTGGCGCGCCCACCCTTTTCAAACAG	
nad2-1F	TTTTTAGCGGTTTCCCCAGAGA	Test *nad2* intron 1 splicing efficiency by RT-PCR
nad2-1R	GGCTTCCGTGGAAAATTCAGAC	
nad2-2F	TGGCGCACCTCTCCTAACTATT	Test *nad2* intron 2 splicing efficiency by RT-PCR
nad2-2R	CGAGCACCAGTGATTTCGTATC	
nad2-3F	CGGATACGAAATCACTGGTGCT	Test *nad2* intron 3 splicing efficiency by RT-PCR
nad2-3R	ACTATGGCGAATGCATCTATCG	
nad2-4F	CTATTGTTGCTTCCTATGGGGG	Test *nad2* intron 4 splicing efficiency by RT-PCR
nad2-4R	GAACAAGGGAGAGGGATATGGA	
nad7-1F	CAGGTGGGACAAGCTCTAGG	Test *nad7* intron 1 splicing efficiency by RT-PCR
nad7-1R	CTCGATTAATTTCTCAGTCCCTC	
nad7-2F	GAGGGACTGAGAAATTAATCGAG	Test *nad7* intron 2 splicing efficiency by RT-PCR
nad7-2R	CTCGACATAAGCCAAGAGGC	
nad7-3F	GCCTCTTGGCTTATGTCGAG	Test *nad7* intron 3 splicing efficiency by RT-PCR
nad7-3R	CCGAACACTTTGTCGCATCT	
nad7-4F	AGATGCGACAAAGTGTTCGG	Test *nad7* intron 4 splicing efficiency by RT-PCR
nad7-4R	GTTTTGGCTCGCAATAAAGC	
EF1α-F	TCTCAAGAACGGTGATGCTG	maize RNA normalization
EF1α-R	TGGGTCCTTCTTCTCCACAC	
nad1-int1F	TGTCAGATCCGAACATAGGG	Test spliced *nad1* exon1-2
nad1-int1R	TGCAGATCGTAATGCTCCTAGA	
nad1-exonF1	TATGTTAAGTCTGGTCGCTTGGG	Test unspliced *nad1* exon1-int1
nad1-intronR1	TATATCATAGGCGACCGAACGG	
nad1-int2F	TGCAGCTCAAATGGTCTCTT	Test spliced *nad1* exon2-3
nad1-int2R	AATACGGGGAACAAGGGAAT	
nad1-exonF2	TCGAAATATGCCTTTCTAGGAG	Test unspliced *nad1* exon2-int2
nad1-intronR2	AAAACTCAAAACGAGCCTTGCG	
nad1-int3F	ATTCCCTTGTTCCCCGTATT	Test spliced *nad1* exon3-4
nad1-int3R	AAAAGAGCAGACCCCATTGA	
nad1-exonF3	GTCATGGCGCAAAAGCAGATATGG	Test unspliced *nad1* exon3-int3
nad1-intronR3	GAATGAGTCCCGAGACATTGGC	
nad1-int4F	TCCCCGTATTGGTTATGTTCC	Test spliced *nad1* exon4-5
nad1-int4R	GATCATATTGGCATACTCTCCC	
nad1-exonF4	GGGAGAGTATGCCAATATGATCTTA	Test unspliced *nad1* exon4-int4
nad1-intronR4	GAGTCAAAGGGTCACCACTACTGAG	
nad2-int1F	AGTAATGTGGGTTGGCTTGG	Test spliced *nad2* exon1-2
nad2-int1R	GAAATGGTACCAGCCGTACTT	
nad2-exonF1	GCGGTTTCCCCAGAGATCTTTC	Test unspliced *nad2* exon1-int1
nad2-intronR1	TACGATTAGCCAGCCTTGCGGC	
nad2-int2F	TCGCAGCATCAAAAAGAAAG	Test spliced *nad2* exon2-3

nad2-int2R	GATCGAAGTGGGTAGCTCCA	Test spliced *nad2* exon2-3
nad2-exonF2	TGATCTTAGGTGCATTTCCCTCTG	Test unspliced *nad2* exon2-int2
nad2-intronR2	ATCGGTAGTAGTCCGGTCGCAC	
nad2-int3F	ACCGGATACGAAATCACTGG	Test spliced *nad2* exon3-4
nad2-int3R	GCGCAATAGAAAGGAATGCT	
nad2-exonF3	TCTACTGGAGCTACCCACTTCGA	Test unspliced *nad2* exon3-int3
nad2-intronR3	AGCGGTACCACCCATCCTACC	
nad2-int4F	GGTTGTGGGGCTTACTTCCT	Test spliced *nad2* exon4-5
nad2-int4R	CGACTTGTCACGATCCATTG	
nad2-exonF4	TTCCAGCATTACGGCAAACC	Test unspliced *nad2* exon4-int4
nad2-intronR4	TACTCATGGCAACCTTCCGGC	
nad4-int1F	GGTGGTTCCTGTTTGGAGAA	Test spliced *nad4* exon1-2
nad4-int1R	AGCGTGCCAATCCCTATGT	
nad4-exonF1	ATGATCGCCGTGTCCTGCATGC	Test unspliced *nad4* exon1-int1
nad4-intronR1	AAGCTTCGCGGGGACCTTGAC	
nad4-int2F	GAAGATCATTGCCTACTCCTCA	Test spliced *nad4* exon2-3
nad4-int2R	AGGGCTGAAGAAACCAGTCC	
nad4-exonF2	CAGTAGCCCATATGAATTTGGTG	Test unspliced *nad4* exon2-int2
nad4-intronR2	CGCTAAGGGGTTTTGTTTTAGG	
nad4-int3F	GTGAACACCCATCCGAACA	Test spliced *nad4* exon3-4
nad4-int3R	GGCGTATTCCCTTTGGCTAT	
nad4-exonF3	TACCCGGCACTAGCAGCTTTATC	Test unspliced *nad4* exon3-int3
nad4-intronR3	CCCATCGCAAGCACCTACAATG	
nad5-int1F	CCATGGATCTCATCGGAAAT	Test spliced *nad5* exon1-2
nad5-int1R	CACATAAATCGAGGGCTATGC	
nad5-exonF1	ATCTCAGAATAGCTCCATGGATCTC	Test unspliced *nad5* exon1-int1
nad5-intronR1	CGGGAGTTGTTACGTCCAGTATG	
nad5-int2F	TTTGCTTTCTGGTTGGGAAG	Test spliced *nad5* exon2-3
nad5-int2R	TCATATCTTTGGCCAAGTATCCTAC	
nad5-exonF2	AGAGCTCGCTTACACAAAGTATACC	Test unspliced *nad5* exon2-int2
nad5-intronR2	TACTTACTTATGGGCTAACAGGTCAC	
nad5-int3F	GATTGGTTTAGGTACAATTTTTGG	Test spliced *nad5* exon3-4
nad5-int3R	TTTGAAAGGCTCGTTGGAAT	
nad5-exonF3	GATATGATGATTGGTTTAGGT	Test unspliced *nad5* exon3-int3
nad5-intronR3	TTTTCCTCAGTTGCAGGGTTTG	
nad5-int4F	CGTACACATTCCGACGATTG	Test spliced *nad5* exon4-5
nad5-int4R	CCCACATACGAGAAAAGGTCA	
nad5-exonF4	AAGGGTGCTATTGAGATATTGGG	Test unspliced *nad5* exon4-int4
nad5-intronR4	CTTTCCTCGGGTTCGTAGAGTC	
nad7-int1F	CGGGCAAATCAAGAATTTCA	Test spliced *nad7* exon1-2
nad7-int1R	CTCGATTAATTTCTCAGTCCCTCT	
nad7-intronF1	GGATTTGCGAATGAATGCTG	Test unspliced *nad7* int1-exon2
nad7-exonR2	CTCGATTAATTTCTCAGTCCCTC	
nad7-int2F	TCAAGCTTTACCTTATTTTGATCG	Test spliced *nad7* exon2-3
nad7-int2R	TGATGCTCCCACATCCATAG	
nad7-intronF2	GTTGTTCGTTCCGTCGTTGA	Test unspliced *nad7* int2-exon3
nad7-exonR3	CTCGACATAAGCCAAGAGGC	
nad7-int3F	GATTGGGGATTCAGTGGTGT	Test spliced *nad7* exon3-4
nad7-int3R	CGAACACTTTGTCGCATCTC	
nad7-exonF3	GCCTCTTGGCTTATGTCGAGATA	Test unspliced *nad7* exon3-int3
nad7-intronR3	ATGGGAACTTCCCCCATATTGC	
nad7-int4F	CCATCACGATCTCGAATGAA	Test spliced *nad7* exon4-5
nad7-int4R	TAGGTGCTTCAACTGCGGTA	
nad7-exonF4	AGATGCGACAAAGTGTTCGGAT	Test unspliced *nad7* exon4-int4
nad7-intronR4	TTTACTCCTAACCCCACGACGG	

ccmFc-F2	TTATTTCGTTTCGTTCCCGTTC	Test spliced *ccmFc* exon1-2
ccmFc-R2	TGTTCAAACATGAACCTTTCGC	
ccmFc-exonF1	CGACTGTTGATGGCTGTTGGTC	Test unspliced *ccmFc* exon1-int1
ccmFc-intronR1	GTCAACTGAGCATCTCAGCGGC	
cox2-int1F	CTCAATGGACGGGGTATTAG	Test spliced *cox2* exon1-2
cox2-int1R	CACAAAGAGCGATTGTGAGG	
cox2-exonF1	AGCTATTGGACATCAATGGTATCG	Test unspliced *cox2* exon1-int1
cox2-intronR1	CGGGGTATAGGTCTAACCACCTC	
rps3-sense	CAGATCCAAGTCGGTTCAGTGA	Test spliced *rps3* exon1-2
rps3-antisense2	AGTCTCGTAGGTGGACGTATCG	
rps3-exonF1	TTTCGGTAAGACTTGATCTGAATCG	Test unspliced *rps3* exon1-int1
rps3-intronR1	CTTTCACGACATGCTCTGGTCC	
14-F5	CATATGAGCCCGTACGCCTCC	Construct Y2H vectors of PPR14
14-R4	CGGATCCCTCATCATTCAAACAGTG	
14-R5	GCGGATCCCGAGGAGGTGCTGCG	
14-F6	CGCCATATGAGCTGCTACGTCTCCC	
14-F7	GCGGATCCATGCGTCGCTACTG	clone PPR14 to pUC-SPYNE vector
14-R6	CCGCTCGAGTTCAAACAGTGTTTG	
14-F8	CGCGGATCCATCTCCCTTGTCCTC	Construct MBP-PPR14-His vector
14-R7	GCGTCGACTCAATGATGGTGATGGTGATGTTCAAACAGTGTTTG	
91y2b-F|SmaI	CCCGGGGGAGTGGACGGACACGGTG	Construct Y2H vectors of PPR-SMR1
91CDS-R|SalI	CGTCGACTCACCTAGGCATGCCAAGGG	
91NE-R|BamHI	TGGATCCGGGGAAGGTGGCGAGGT	
91P-F|EcoRI	TGAATTCAAGACCTTCAACGCCGTC	
91P-R|BamHI	TGGATCCTGCCTTGGCGAACAGCTTAT	
91CDS-F|BamHI	CGGATCCATGCTGCTCCGCGTTGGC	clone PPR-SMR1 to pUC-SPYCE vector
PPR-SMR1R3	GGCCGACGTCGACCCTAGGCATGCCAAGGG	
91CP-F|BamHI	GGATCCGAGTGGACGGACACGGTG	Construct MBP-PPR-SMR1-His vector
91CH-R|SalI	GTCGACTTAGTGGTGGTGGTGGTGGTGCCTAGGCATGCCAAGGGA	
CSFy2b-F1|EcoRI	GAATTCTACGGCTTCGTGGCCC	Construct Y2H vectors of Zm-mCSF1
CSFy2b-R1|BamHI	GGATCCCTAAATTACTTTTGTAATTTGGCAC	
mCNE-R|BamHI	TGGATCCCAGTACCCGCTCCCGCT	
mCRM-F|EcoRI	TGAATTCGGAGAGCCTCTCACCCC	
mCRM-R|BamHI	TGGATCCATTTCCCCTCCAAACAATGA	
mCCE-F|EcoRI	TGAATTCGAAGATGGGAGCCTACAAG	
mC-F|BamHI	AGGATCCATGCTCACCCTCCCCGGT	clone Zm-mCSF1 to pUC-SPYCE vector
mC-RNS|XhoI	ACTCGAGAATTACTTTTGTAATTTGGCAC	
